# “Grafting‐to” Polymers of Xylan‐*g*‐allyl Glycidyl Ether Toughen PEG Hydrogel via Microphase Separation: Thermoresponsive and Photoreactive Molecular Assembly in DLP 3D Printing

**DOI:** 10.1002/smll.202502129

**Published:** 2025-07-01

**Authors:** Yidong Zhang, Qingbo Wang, Wangfang Deng, Silva Hazer, Axel Luukkonen, Andrey Pranovich, Outi M. H. Salo‐Ahen, Ronald Österbacka, Chunlin Xu, Xiaoju Wang

**Affiliations:** ^1^ Laboratory of Natural Materials Technology Åbo Akademi University Henrikinkatu 2 Turku FI‐20500 Finland; ^2^ Pharmaceutical Sciences Laboratory Åbo Akademi University Tykistökatu 6A Turku FI‐20520 Finland; ^3^ Structural Bioinformatics Laboratory Åbo Akademi University Tykistökatu 6A Turku FI‐20520 Finland; ^4^ Physics, Åbo Akademi University Henrikinkatu 2 Turku FI‐20500 Finland

**Keywords:** conductive hydrogel, DLP printing, photopolymerization, thermoresponsive polymer, xylan

## Abstract

Utilizing naturally derived biopolymers in the macromolecular design of thermoresponsive polymers offers sustainable and biodegradable smart building blocks to functional materials. Here, a novel graft polymer of xylan‐*g*‐allyl glycidyl ether (xylan‐*g*‐AGE) that is thermoresponsive to self‐assemble and photoreactive in photopolymerization is reported. This research highlights an innovative use of the debranched wood xylan, a chemically engineered linear polysaccharide of *β*‐1,4‐linked xylose, as the backbone in grafting polymer, which allows a greater degree of spatial coordination for sidechains than the analogous cellulose. Induced by the reformation of H‐bonds and hydrophobic effect, xylan‐*g*‐AGE transits from solvated coil chain to self‐assembled mesoglobules upon the temperature change above its lower critical solution temperature (LCST). When xylan‐*g*‐AGE is used in photoresins to fabricate hydrogels with good geometric fidelity via DLP 3D printing, solvated xylan‐*g*‐AGE stiffens the polyethylene glycol (PEG) hydrogel strongly, due to higher crosslink density of available AGE moiety and faster crosslinking rate, while self‐assembled xylan‐*g*‐AGE toughens the PEG hydrogel better, attributed to the strategy of “dual chemically independent domains” that smartly combines tough domain of PEG and soft domain of self‐assembled xylan‐*g*‐AGE. Conductive hydrogel is fabricated by incorporating 2D MXene sheets into this hydrogel matrix in DLP printing, which demonstrates superior performance as wearable strain sensors.

## Graphical abstract

A novel thermoresponsive and photoreactive xylan‐*g*‐allyl glycidyl ether (xylan‐*g*‐AGE) is constructed by the grafting modification of AGE to debranched xylan (D‐xylan). Benefiting from the greater conformational flexibility of D‐xylan, xylan‐g‐AGE can transit from solvated coil chain to self‐assembled mesoglobule upon temperature increases above the lower critical solution temperature. Xylan‐*g*‐AGE toughens PEG hydrogel via microphase separation, benefiting the high geometric fidelity in DLP 3D printing and fabrication of conductive MXene strain sensors.

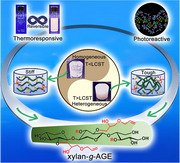

## 1 Introduction

Thermoresponsive polymers are among the most extensively investigated smart building blocks for designing functional materials.^[^ ^1^ ^]^ These polymers can undergo conformational or phase transitions in response to variations in temperature, which simultaneously results in the assembly of macromolecules to construct diverse nanostructures with unique morphology and size.^[^ ^2^ ^]^ These thermoresponsive polymer assemblies are thus of interest for enabling precise control over material topology in the construction of topologically controlled structures. Moreover, the temperature‐responsive phase transition of macromolecules provides an external mechanism to conveniently induce the microstructure evolution in biphasic systems, thereby influencing overall material properties (e.g., topography and mechanics) and generating new functional materials.^[^ ^3^ ^]^ Till now, the design and implementation of precisely controlled assembly has been mainly achieved with synthetic polymers that show thermal responsive characteristics of phase transition, in which important families of poly(N‐substituted acrylamide)s (e.g., poly(N‐isopropylacrylamide)) and poly(alkyoxide)s (e.g., poly(oligo(ethylene glycol) methacrylate)) are exemplified.^[^ ^4^ ^]^ To align with the societal transformation toward sustainability, macromolecular design and synthesis of biodegradable and naturally derived biocompatible thermoresponsive polymers have garnered attention, and interest in their utilization has grown in recent years. Natural polysaccharides are pivotal candidates in this green materials revolution.^[^ ^5^ ^]^ Among the synthetic routes, the “grafting to” approach to tether sidechains to each repeating unit of a linear polymer backbone (typically A‐*g*‐B) has been seen effective in configuring the polysaccharides biopolymer with grafted hydrophobic sidechains to impart thermal responsiveness.^[^ ^5^ ^]^ In this context, the sidechain length and grafting density, together with the hydrophobicity in the sidechain segments, are the most relevant to the molecular design of graft polymers. The substitution of hydrophilic groups (─OH) of polysaccharides (e.g., cellulose, starch, and dextran) by hydrophobic sidechains (e.g., methoxy‐, alkyl‐, alkyl amide‐, and poly(*N*‐vinylcaprolactam)‐) could weaken the hydrogen bonds between water and hydroxyl groups, leading to thermoresponsive self‐assembly behaviors through hydrophobic interactions.^[^ ^6^ ^]^

**Scheme 1:**
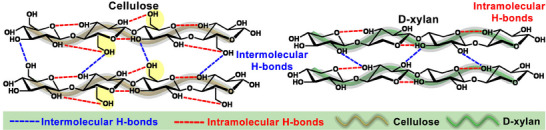
Comparison of the intra‐/intermolecular H‐bonds between different polysaccharide chains.

Xylan is the third most abundant renewable polysaccharide in nature after cellulose and chitin, which shares the same β‐1,4 glycosidic linkage as these polysaccharides, but consists of maintenance of *β*‐1,4‐linked xylose with side branches of *α*‐arabinofuranose and/or *α*‐glucuronic acids.^[^ ^7^ ^]^ In contrast to the crystalline structure of linear cellulose and chitin, native xylan is an amorphous, acetylated, and branched biopolymer.^[^ ^8^ ^]^ Notably, the acetyl groups and side uronic acid units of xylan could interrupt the inter‐/intramolecular hydrogen bonds (H‐bonds) among the macromolecules of xylan itself, thereby impairing the close packing of xylan chains.^[^ ^9^ ^]^ Hence, the extreme level of sidechain elimination is indispensable for the close packing of xylan chains. From this perspective, we recently have reported an engineering approach to produce almost linear/debranched xylan (D‐xylan of *β*‐1,4‐linked xylose), by alkaline treatment under the reformation of the reducing‐end group by borohydride reduction.^[^ ^10^ ^]^ D‐xylan is insoluble in water but can be dissolved in highly alkaline solution (3 m NaOH) at room temperature. When regenerated from alkaline solution via pH shifting, D‐xylan was seen to self‐assemble as primary crystallites (10–15 nm) of cubic morphology.^[^ ^10^ ^]^ At the level of macromolecular conformation, cellulose maintains a rigid twofold helical screw conformation attributed to the extensive intra‐/intermolecular H‐bonds inherent to each repeating unit (Scheme 1).^[^ ^11^ ^]^ In comparison to cellulose, the absence of exocyclic CH_2_OH on C5 in D‐xylan reduces the intramolecular H‐bonds, simultaneously endowing a broader conformational space of glycosidic dihedral angles.^[^ ^12^ ^]^ D‐xylan is inferred by molecular dynamics (MD) simulations to form a more flexible threefold helical screw conformation in water, i.e., one 360° twist per three glycosidic bonds (Scheme 1).^[^ ^13^ ^]^ For constructing the molecular assembly of thermoresponsive polymers, linear D‐xylan is thus hypothesized to provide a greater conformational flexibility to spatially coordinate the side chains than cellulose, for instance.

The self‐assembly behaviors of graft polymers are determined by the tunable structural parameters, including the dimensions of the backbone and side chain, grafting density, and grafting length. The engineered biopolymer of D‐xylan serves as a linear backbone of *β*‐1,4‐linked xylose units for the synthesis of a graft polymer, with even accessibility to react with hydroxyl groups (C2‐ and C3‐OHs) in D‐xylan. We proposed the grafting of allyl glycidyl ether (AGE) to D‐xylan for preparing a thermoresponsive biopolymer, due to the highly stable ether bond between D‐xylan and AGE, and the hydrophobicity of the terminated end groups in the AGE moiety. We anticipated that designing xylan‐based biopolymer grafted with hydrophobic AGEs could interrupt the inter‐/intramolecular H‐bonds among the macromolecules of D‐xylan itself and subsequently enhance solubility in water. Meanwhile, it is expected that the introduction of hydrophobic short‐carbon sidechains may contribute to the hydrophobic effect upon the temperature variation, driven by the multi‐factor intermolecular interactions: i) van der Waals forces and H‐bonds, and ii) hydrophobic interactions among the AGE sidechains. To this end, we were then inspired to investigate the thermoresponsive property of xylan‐*g*‐allyl glycidyl ether (xylan‐*g*‐AGE) and its molecular assemblies driven by temperature change in solution and using MD simulations. To the best of the authors’ knowledge, no xylan‐based thermoresponsive polymers have been reported to date.

Lithography‐based digital light processing (DLP) 3D printing has gained prominence as an additive manufacturing (AM) approach for fabricating nanocomposite hydrogels due to high optical resolution (1–50 µm), rapid printing speed, and unparalleled accuracy in microarchitecture.^[^ ^14^ ^]^ The photoreactivity of xylan‐*g*‐AGE, conferred by the photopolymerizable ene‐ending group in AGE, creates opportunities to integrate this biopolymer and its assemblies as macromonomers for hydrogel fabrication using DLP technology. This initiative also addresses the critical need for developing sustainable photopolymers and bio‐derived photoresin in vat 3D printing. Achieving robust mechanics is essential for ensuring high printing fidelity in DLP 3D printing of hydrogel objects.^[^ ^15^ ^]^ Special material design strategies such as well‐aligned nano‐/microstructures, double networks, fibrous scaffolds, and supramolecular interactions are commonly used to reinforce the hydrogel network during the printing workflow, which improves the mechanical parameters (e.g., stiffness and toughness) and dissipates energy when the hydrogel is undergoing deformation.^[^ ^16^ ^]^ In this regard, we employed thiol‐ene “click” chemistry to improve the homogeneity of the resulting hydrogel networks, utilizing macromonomers such as xylan‐*g*‐AGE, poly(ethylene glycol) diacrylate (PEGDA), and 4‐arm poly(ethylene glycol) thiol (PEGSH). Importantly, material nanoconstruction and bicontinuous morphology creation have been proven effective for enhancing the hydrogel's mechanical properties.^[^ ^17^ ^]^ The thermoresponsiveness of xylan‐*g*‐AGE enables delicate control over microphase separation, potentially resulting in a biphasic distribution of xylan and polyethylene glycol (PEG) within the hydrogel precursor system. Upon the photopolymerization in the vat, the phase transition in response to temperature control is hypothesized to create dual, chemically independent domains of molecular assembly of xylan‐*g*‐AGE and PEG network, which are, nevertheless, covalently bonded to form the polymeric networks in hydrogels. This method is aligned with the reinforcement strategy of nano‐/microconsctruction through a temperature‐induced phase separation (*TIPS*) technique, and we have thus aimed to toughen the hydrogel network by integrating these smart molecular assemblies of xylan in DLP printing. To further demonstrate the extendable possibility of our photoresins, we continue to validate the fabrication of MXene‐containing composite hydrogels via DLP 3D printing, aiming at high printing fidelity. After the typical etching and delamination process of the MAX phase, the MXene sheet possesses highly active surface groups (e.g., ─OH, ─F, and ─O) with high hydrophilicity, guaranteeing high dispersibility of MXene in water. Hence, it is expected that xylan‐*g*‐AGE with O‐rich functional groups could enhance the dispersion of MXene in hydrogels. When MXene sheets are encapsulated in the photoresins, the intrinsic light‐scattering challenge for DLP printing might be amplified by the nano‐effect to cause limited light penetration and incomplete curing.^[^ ^18^ ^]^ In this regard, it is revealed that the fast crosslinking by “click” thiol‐ene photopolymerization is critical to fabricate the MXene‐containing composite hydrogel with our photoresin. To this end, we have successfully produced wearable and portable electronics using the above MXene composite hydrogel, which is not only sensitive to strain variation but also intrinsically flexible and stretchable to accommodate significant deformations in use.

## 2 Results and Discussion

### 2.1 Material Design Rationale

**Figure 1:**
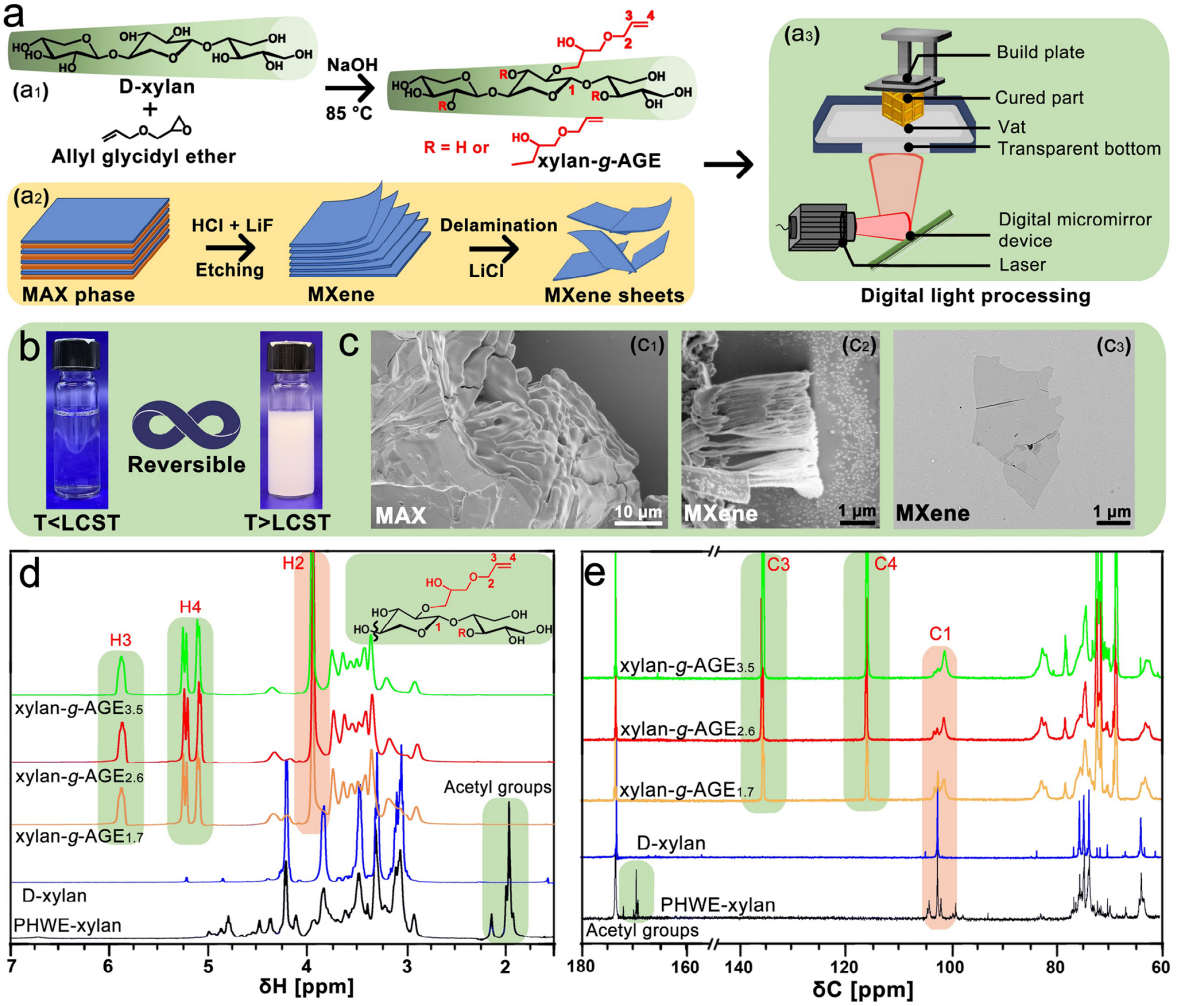
Material design rationale and characterization. a_1_) Synthesis of xylan‐g‐AGE, a_2_) preparation of MXene sheets using HCl‐LiF etching and LiCl delamination, and a_3_) DLP 3D printing; b) The thermo‐reversible transition of aqueous xylan‐g‐AGE_2.6_; Morphology of c_1_) MAX phase, c_2_) MAX phase after HCl‐LiF etching, and c_3_) MXene sheet; d) Liquid state ^1^H and e) quantitative ^13^C NMR spectra of PHWE‐xylan, D‐xylan, and xylan‐*g*‐AGE, demonstrating chemical composition of the modified biopolymer.

The present research has aimed to toughen PEG hydrogel via incorporating thermoresponsive and photopolymerizable xylan*‐g‐*AGE to fabricate conductive hydrogels for potential applications in wearable sensors via DLP 3D printing technology. An overview of the engineering of building blocks and the fabrication strategy is proposed in **Figure** **1**a, which involves the grafting modification of AGE to D‐xylan, the preparation of MXene sheets, and DLP printing. In the grafting reaction, D‐xylan as the starting material was reacted with AGE via base‐catalyzed etherification of the ─OH with an oxirane group from AGE, and subsequently dialyzed against water to form water‐soluble xylan‐*g*‐AGE (Figure 1a_1_). This modification imparts the free‐radical activity and hydrophobic functionality to the biopolymer. Generally, thermoresponsive polymers are divided into two categories: polymers that exhibit a lower critical solution temperature (LCST) or an upper critical solution temperature (UCST), and this reversible phase transition is determined by the so‐called “hydrophobic effect” of water.^[^ ^19^ ^]^ The xylan‐*g*‐AGE changed from a dissolved homogeneous state to a two‐phase demixed system as the temperature increased, indicating the LCST performance (Figure 1b). Then, PEGDA hydrogel was toughened by incorporating the thermoresponsive xylan‐*g*‐AGE either as a water‐soluble macromonomer below the LCST or as molecular self‐assemblies above the LCST. Meanwhile, a synthesis protocol of HCl‐LiF etching, LiCl delamination, and post‐delamination processing by centrifugation and ultrasonication was used to prepare high‐quality MXene sheets (Figure 1a_2_). Compared to the close‐packed nanolaminated structure of the unreacted MAX phase (Figure 1c_1_), well‐spaced layered structure (Figure 1c_2_) was yielded due to the expansion of MAX phase when the Al layer was etched, ultimately forming single‐to‐few layered MXene sheets with a size of ≈2–3 µm (Figure 1c_3_). MXene was composited in toughened PEGDA hydrogel to showcase the potential application as conductive hydrogels. Finally, high‐fidelity hydrogels with controlled internal structures were 3D printed by DLP, which manufactures hydrogels in a layer‐by‐layer approach using a digital micromirror device to direct the projected light and to photopolymerize liquid photoresins, as shown in Figure 1a, _3_.

### 2.2 Thermoresponsive Graft Polymer of xylan‐*g*‐AGE and its Self‐Assemblies: Synthesis and Characterizations

As displayed in Figure 1d,e, the chemical structure and degree of substitution (DS) of xylan‐*g*‐AGE were measured by liquid state ^1^H and quantitative ^13^C NMR. For the starting materials, the peaks at 1.9 ppm in the ^1^H (Figure 1d) spectra and 169 ppm in the ^13^C spectra (Figure 1e) were ascribed to the acetyl groups of the pressurized hot water extracted xylan (PHWE‐xylan),^[^ ^20^ ^]^ whereas the signals disappeared in the D‐xylan samples due to the deacetylation/debranching treatment. After reaction with AGE, the new emerging signal at 5.8 ppm (H3), and the split peaks at 5.1 and 5.2 ppm (H4) belong to three unsaturated vinylene protons.^[^ ^21^ ^]^ Additionally, the peak at 4.0 ppm was ascribed to the methylene protons connected to the vinyl group (H2).^[^ ^22^ ^]^ These results indicate that AGE molecules were successfully grafted onto the D‐xylan backbone. Liquid state ^13^C NMR was further used to quantify the DS of xylan‐*g*‐AGE (Figure 1e). The new peaks at 116.1 (C3) and 136.1 ppm (C4) are ascribed to the vinyl groups of xylan‐*g*‐AGE, implying the etherification success between the ─OH of D‐xylan and epoxy groups of AGE.^[^ ^21^ ^]^ The DS of xylan‐*g*‐AGE was calculated by comparing the integration of C═C double peaks (116.1 and 136.1 ppm) to anomeric carbons (C1, 101.5–103.4 ppm) as the ratio of A_AGE_ (116.1 + 136.1 ppm)/2A_C1_ (102 ppm). Theoretically, the full DS of xylan is 2, implying that AGE molecules have been grafted onto all the C2‐ and C3–OHs in anhydrosugar units. As presented in Figure 1e, the DS of xylan‐*g*‐AGE gradually increased from 1.7 to 3.5 with the increase of AGE, which was higher than that of other reported values, e.g., DS = 2.5 for *n*‐butyl glycidyl ether grafted onto galactoglucomannan (GGM) and DS = 0.83 for AGE grafted onto GGM.^[^ ^20^, ^21^ ^]^ This result was due to the high amount of NaOH (5.5 wt.%) and the high molar ratio of xylan: AGE (1:3–1:7) deployed in the current synthesis. AGE forms a new ─OH from the epoxide ring‐opening reaction during the etherification reaction, which serves as a potential reaction site for unreacted AGE molecules, as shown in Figure S1 (Supporting Information), leading to the higher DS of xylan‐*g*‐AGE than the theoretical value (DS = 2). However, compared to xylan‐*g*‐AGE_2.6_ with the D‐xylan as the starting material, the grafting reaction using the PHWE‐xylan under the same reaction conditions only yielded a much lower DS (≈1.8) for AGE‐PHWE‐xylan, as shown in Figure S2 (Supporting Information). This result strongly indicates that side groups of PHWE‐xylan potentially limit the accessibility of the reaction sites and impede the grafting of AGE with a full substitution onto xylan.

As shown in Figure S3 (Supporting Information), the peaks at 1725 cm^−1^ can be ascribed to C═O carbonyl stretching vibrations of acetyl groups of xylan^[^ ^23^ ^]^ but were hardly detectable in the D‐xylan after deacetylation treatment. The new peak at 1643 cm^−1^ was attributed to the etherified modification of AGE onto D‐xylan main chains.^[^ ^24^ ^]^ With the increase of DS, the peaks of ─OH of xylan‐*g*‐AGE shift to higher wavenumbers (from 3405 to 3444 cm^−1^), due to the difference between the new ─OH bonds in AGE molecules and ─OH bonds in D‐xylan main chains.^[^ ^20b^ ^]^ The average molecular weight (Mw) and the number average molecular weight (Mn) of xylan‐*g*‐AGE increased after the AGE modification, e.g., the Mw of xylan‐*g*‐AGE increased from 5.6 to 17.5 kDa, and the Mn increased from 4.1 to 7.2 kDa (Figure S4, Supporting Information). The onset degradation temperature (*T_o_*) and maximum degradation temperature (*T_max_*) of xylan‐*g*‐AGE were much higher than those of D‐xylan and PHWE‐xylan, ascribed to the successful grafting of AGE onto the polymer main chain (Figure S5 and Table S1, Supporting Information). The glass transition temperature (*Tg*) is the typical temperature of an amorphous polymer (190.6 °C for PHWE‐xylan), while the *Tg* of D‐xylan was hardly detectable, due to the increased *CrI* and enhanced H‐bonds network of D‐xylan after debranching and deacetylation (Figure S6, Supporting Information), which could restrict the mobility of polymer chains. However, *Tg* was observed for all xylan‐*g*‐AGEs in various DS, which are much lower than that of PHWE‐xylan, and furthermore, the *Tg* of xylan‐*g*‐AGE decreased from 72.2 to 50.6 °C as the DS increased from 1.7 to 3.5, probably due to the increased molecular mobility of the grafted AGE moieties along the xylan backbone.

**Figure 2:**
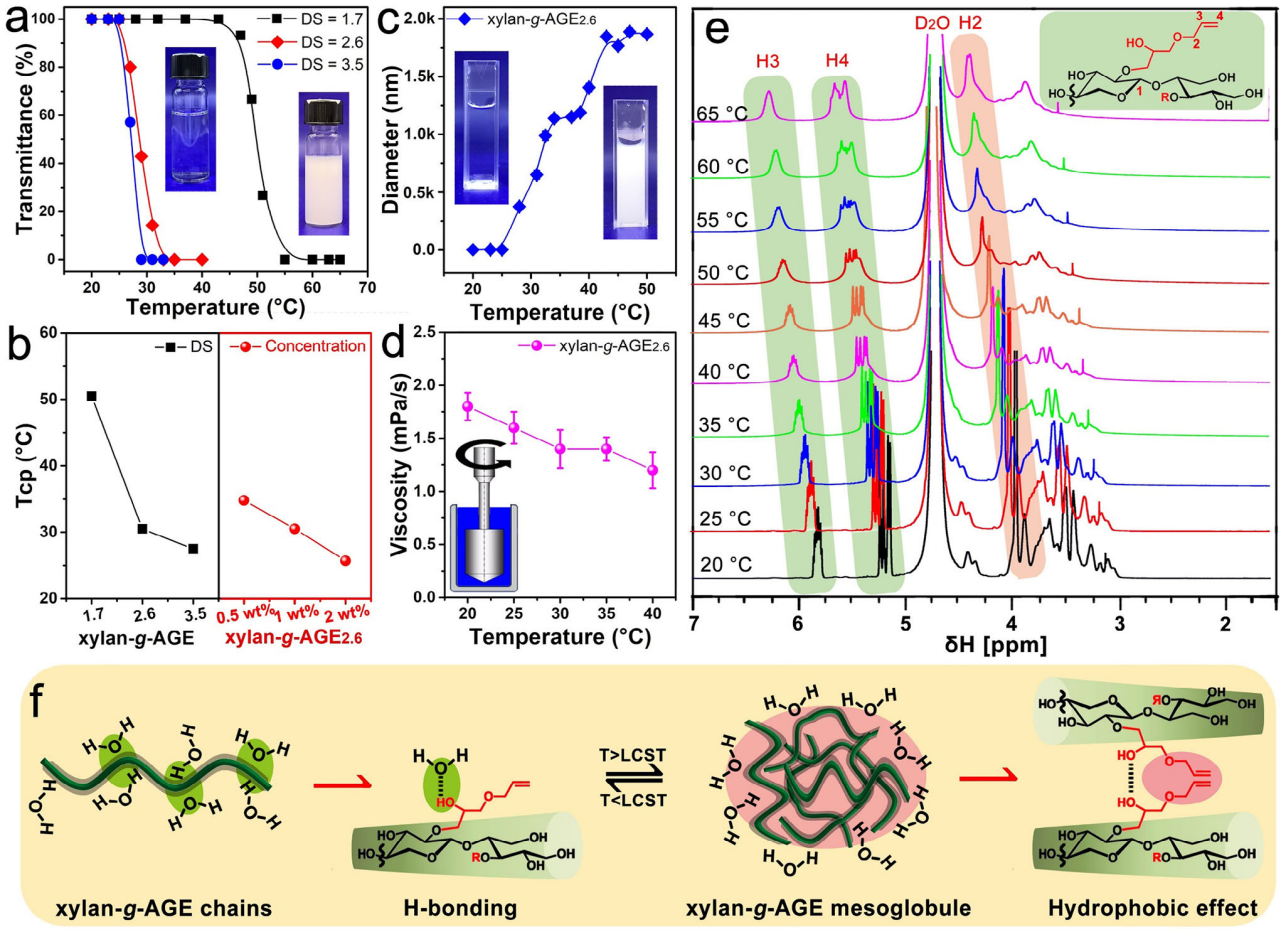
Physiochemical properties of xylan‐*g*‐AGE. a) Diagram of transmittance versus temperature of an aqueous xylan‐*g*‐AGE with full transmittance below the LCST followed by complete suppression above the LCST; b) *T_cp_* of xylan‐*g*‐AGE in different DS and xylan‐*g*‐AGE_2.6_ at various concentrations; c) Diagram of hydrodynamic diameter versus temperature of xylan‐*g*‐AGE_2.6_ at different temperatures; d) Viscosity versus temperature of xylan‐*g*‐AGE_2.6_ at various temperatures; e) ^1^H NMR spectra of thermoresponsive xylan‐*g*‐AGE in D_2_O below and above LCST; f) Schematic illustration on the transition of xylan‐*g*‐AGE solvated random chain to insoluble mesoglobule transition with well polymer solvation below the LCST and domination of the hydrophobic interactions above the LCST.

For practical and specific applications, the cloud point temperature (*T_cp_*) or LCST of thermoresponsive polymers that is tunable over a wide window is highly required. **Figures** **2**a and S7 (Supporting Information) show that xylan‐*g*‐AGE exhibited sharp and reversible phase transitions as the temperature changed. Additionally, the sharpness of the phase transition diminished as the DS of xylan‐*g*‐AGE decreased, due to the weakened effect of syneresis. The LCST was recorded as the temperature at which the sample's transmittance was 50%, and the *T_cp_* of xylan‐*g*‐AGE_1.7_, xylan‐*g*‐AGE_2.6_, and xylan‐*g*‐AGE_3.5_ was 50.5, 30.5, and 27.5 °C, respectively (Figure 2b). Taking account of the convenience of temperature control, xylan‐*g*‐AGE_2.6_ was selected to construct the thermoresponsive hydrogel, and the *T_cp_* of xylan‐*g*‐AGE_2.6_ at different concentrations was detected as well, as shown in Figure S8 (Supporting Information). The *T_cp_* of xylan‐*g*‐AGE_2.6_ at concentrations of 0.5 wt.%, 1 wt.%, and 2 wt.% was 34.8, 30.5, and 25.7 °C, respectively, and increasing the concentration of xylan‐*g*‐AGE increases the effect of syneresis, and vice versa (Figure 2b). These results suggest that the LCST of xylan‐*g*‐AGE could be tailored by controlling the DS or concentration of xylan‐*g*‐AGE. In contrast, AGE‐PHWE‐xylan presented gentle and slow phase transitions as the temperature increased from 20 to 65 °C (Figure S9, Supporting Information), ascribed to the weakened hydrophobic interactions between AGE‐PHWE‐xylan chains with respect to those in xylan‐*g*‐AGE_2.6_. This indicates that the presence of side groups in native xylan might also impair the reformation of H‐bonds and hydrophobic interactions, leading to gentle and slow phase transitions upon temperature change. Moreover, the phase transitions of xylan‐*g*‐AGE can be triggered by only hand temperature (Video S1, Supporting Information), indicating the potential for biomedical applications. Dynamic light scattering (DLS) technique was used to examine the thermoresponsive behavior of xylan‐*g*‐AGE_2.6_ and AGE‐PHWE‐xylan at 10 mg mL^−1^ with temperatures varying from 20 to 50 °C at an increment of 3 °C (Figure 2c; Figure S10, Supporting Information). The hydrodynamic radius of xylan‐*g*‐AGE_2.6_ drastically increased from 2 nm below the LCST to 1000 nm above the LCST, indicating that the xylan‐*g*‐AGE presented hydrated coil‐like chains in a clear solution at 25 °C and then formed aggregates (also called mesoglobules) at 35 °C. The hydrodynamic radius of these aggregates continued to increase progressively upon heating above the LCST at 40 °C, reaching a plateau value at ≈1800 nm. However, AGE‐PHWE‐xylan exhibited a relatively slow phase transition/particle size increase, with small particles observed below LCST, indicating that AGE‐PHWE‐xylan cannot be fully dissolved in water (Figure S10, Supporting Information). Moreover, the viscosity of xylan‐*g*‐AGE_2.6_ slightly decreased as the temperature increased from 20 to 40 °C, suggesting that xylan‐*g*‐AGE after LCST has more free water than that below LCST (Figure 2d).

**Figure 3:**
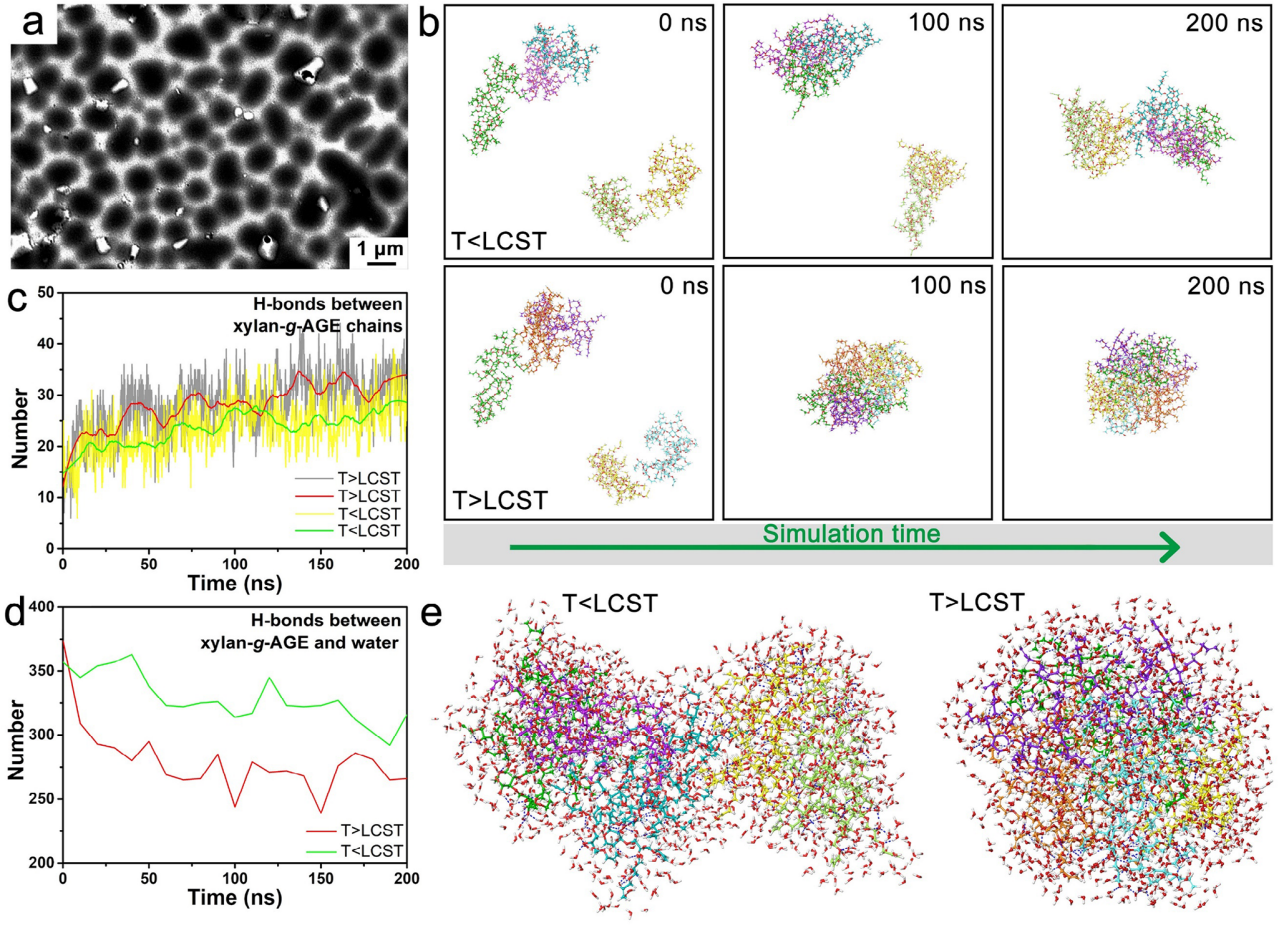
Temperature‐dependent molecular interactions. a) TEM images of the xylan‐*g*‐AGE above LCST; b) Trajectory snapshots of five xylan‐*g*‐AGE chains at different simulation time points of the 200 ns long MD simulation below and above LCST (non‐polar hydrogen atoms and water molecules are hidden for clarity; all polymer chains are shown in different colors; c) Number of intra‐ and intermolecular H‐bonds between xylan‐*g*‐AGE chains during 200 ns MD simulation; d) Number of H‐bonds between xylan‐*g*‐AGE chains and water; e) Snapshot structures of xylan‐*g*‐AGE and interacting water molecule after the MD simulation. Atom color code: red–oxygen; white–hydrogen; carbon–according to the chain color. H‐bonds are shown as blue dashed lines.

In order to investigate, verify, and understand the thermoresponsive behavior of xylan‐*g*‐AGE observed experimentally at the molecular level, we performed ^1^H NMR of xylan‐*g*‐AGE in D_2_O in the range of 20 to 65 °C (Figure 2e) and MD simulations of xylan‐*g*‐AGE chain containing 10 xylose molecules in water below/above LCST (**Figure** **3**). The xylan‐*g*‐AGE chains are well‐hydrated when the temperature is below LCST, leading to sharp peaks in the ^1^H NMR spectrum, whereas the sharp peaks become broadened when the LCST is exceeded, as a result of the grafted AGE decreasing the chain mobility of xylan‐*g*‐AGE and the changes in the H‐bond interaction between xylan‐*g*‐AGE and D_2_O (Figure 2e). Also, the peaks shift to higher fields as the temperature increases, because of the temperature dependence of the H‐bonds. Thus, the change and shift of peaks indicate the change of the hydrated environment of double bonds and the transition of H‐bonds during the reversible phase transitions as the temperature changes. These results were confirmed by the MD simulation analysis (Figure 3). During a 200 ns simulation, different interaction patterns between the polymer chains and water molecules were observed at different simulation temperatures. At the beginning of the simulations (at 0 ns), the xylan‐*g*‐AGE chains were dispersed in water. Above LCST, the polymer chains quickly assembled and formed tightly packed, globular macromolecular aggregates (100 ns, 200 ns) (Figure 3b). The experimental morphology of the mesoglobules that were formed by 1 wt.% xylan‐*g*‐AGE_2.6_ above LCST was imaged by TEM (Figure 3a). In contrast, along with time, only loose aggregates of xylan‐*g*‐AGE with fewer interactions were observed below the LCST (Figure 3b). We further analyzed the number of H‐bonds between xylan‐*g*‐AGE and H_2_O to investigate the factors influencing the inter‐ and intermolecular interactions (Figure 3c,d). Compared to the results below LCST, the number of H‐bonds between xylan‐*g*‐AGE chains increased more above LCST, and H‐bonds between xylan‐*g*‐AGE chains and H_2_O decreased more above LCST, indicating the reorganization of H‐bonds and hydrophobic effect as temperature changed, which induced the change of configuration and self‐assembly behavior of xylan‐*g*‐AGE at the temperature above LCST. Additionally, all signals (including the backbone and side chain) of xylan‐*g*‐AGE remain visible in the ^1^H NMR above the LCST at 65 °C (Figure 2e), indicating that the collapsed xylan‐*g*‐AGE still contains water molecules, which agrees with the simulation results on the interactions between xylan‐*g*‐AGE and water above the LCST (Figure 3e).

Xylan‐*g*‐AGE has exhibited reversible phase transition in response to external temperature changes, and the thermoresponsive behavior of xylan‐*g*‐AGE on the molecular level is illustrated in Figure 2f. Below the LCST, xylan‐*g*‐AGE is soluble due to the extensive H‐bond interactions between xylan‐*g*‐AGE and surrounding water molecules. Above the LCST, water molecules were repelled from the xylan‐*g*‐AGE chain, deconstructing the extensive H‐bond interactions with water molecules (Figure 2f). Meanwhile, the intra‐/intermolecular H‐bonds and hydrophobic effect of double bonds between xylan‐*g*‐AGE chains reformed. The hydrophobic interactions and reformed H‐bonds dominate induced polymer globule formation, eventually resulting in an insoluble phase separation in an aqueous solution upon heating. Thus, the intermolecular interactions between xylan‐*g*‐AGE chains under the influence of temperature are confirmed through *T_cp_*, DLS, NMR, and MD simulations.

### 2.3 Toughening PEG Hydrogel with Thermoresponsive xylan‐*g*‐AGE in Photopolymerization Either as Solvated Macromonomer or as Self‐Assembled Mesoglobules

**Figure 4:**
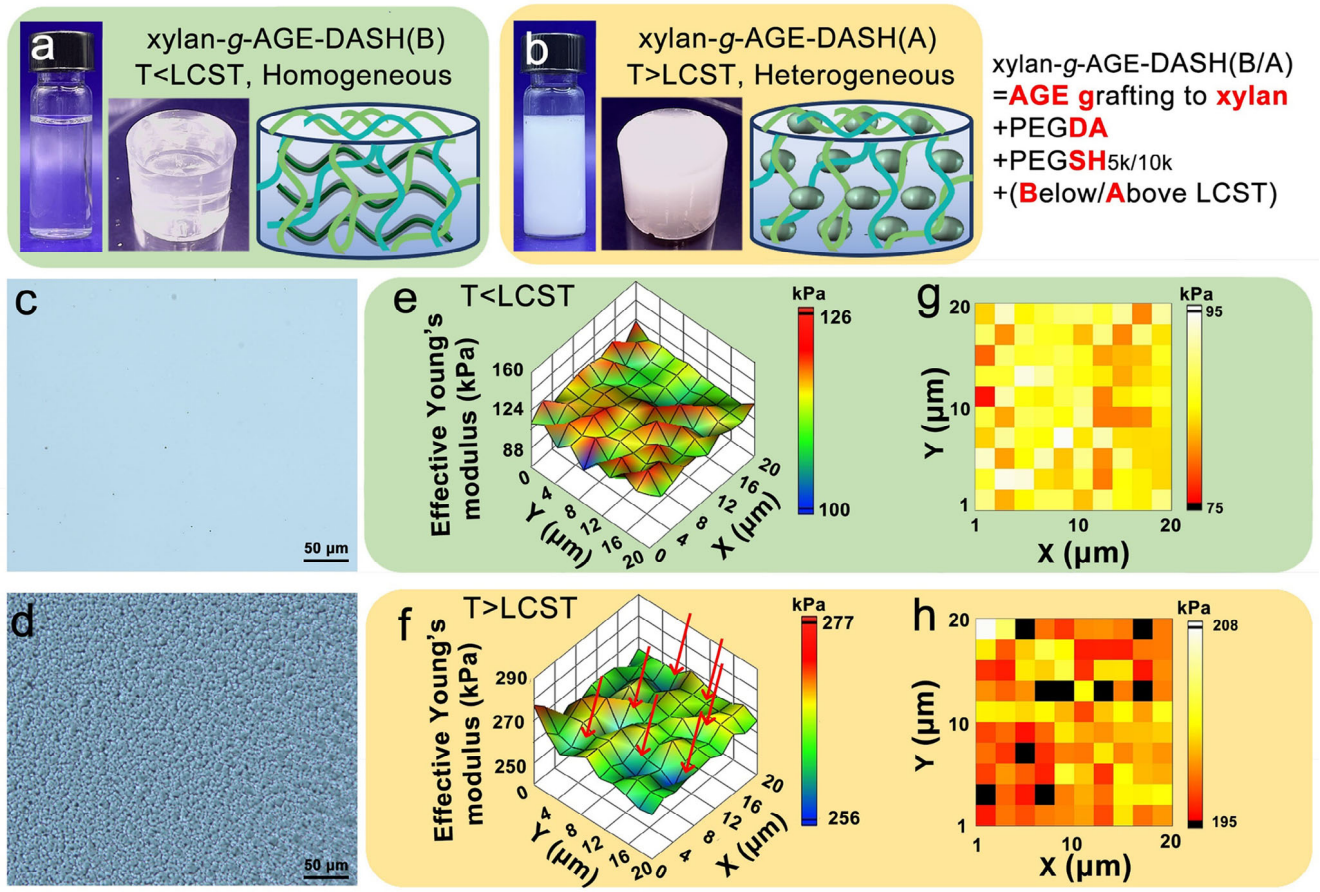
Effect of xylan‐*g*‐AGE on hydrogels prepared by photo‐crosslinking. Photographs of xylan‐*g*‐AGE‐DASH photoresins and hydrogels, and schematic illustration demonstrating a) the homogeneous (xylan‐*g*‐AGE‐DASH(B)) and b) heterogeneous (xylan‐*g*‐AGE‐DASH(A)) hydrogels, where B and A represent hydrogels photo‐crosslinked below or above LCST, respectively; Optical micrographs of xylan‐*g*‐AGE‐DASH photo‐crosslinked c) below and d) above LCST with a height of 100 µm; e,f) Surface effective Young's modulus and g,h) representative heat maps of effective Young's modulus distribution of xylan‐*g*‐AGE‐DASH hydrogel photo‐crosslinked below and above LCST.

Taking the advantage that xylan‐*g*‐AGE is both thermoresponsive and photoreactive, the UV casting process was performed to crosslink xylan‐*g*‐AGE, PEGDA, and 4‐arm PEGSH as a multicomponent photoresin of xylan‐*g*‐AGE‐DASH below/above LCST. As shown in **Figure** **4**a,b, xylan‐*g*‐AGE‐DASH(B) was transparent with the size of 10 × 15 mm (Ø × H) when photocured below LCST, due to the well‐hydrated status of xylan‐*g*‐AGE, while xylan‐*g*‐AGE‐DASH(A) was opaque when photocured above LCST, ascribed to the *TIPS* of xylan‐*g*‐AGE. The distinct morphology between xylan‐*g*‐AGE‐DASH(B) and xylan‐*g*‐AGE‐DASH(A) was captured by optical microscopy imaging of the photocured hydrogels with a thickness ≈100 µm (Figure 4c,d). The phase transition of xylan‐*g*‐AGE from well‐hydrated macromolecular coil‐chains to self‐assembled mesoglobules has resulted in an ordered phase separation of microsized domains, creating the topography and texture as seen for the xylan‐*g*‐AGE‐DASH(A) hydrogel in Figure 4d. Then, we sought to determine if the observed morphology resulted in different mechanical properties of the hydrogel by using nanoindentation. Overall, the xylan‐*g*‐AGE‐DASH(A) exhibited more than twice the effective Young's modulus than xylan‐*g*‐AGE‐DASH(B), as shown in Figure 4e,f. Notably, the hydrogel of xylan‐*g*‐AGE‐DASH(B) exhibited a more uniform distribution of the effective Young's modulus over a mapping of 20 × 20 µm, when compared to xylan‐*g*‐AGE‐DASH(A). In the hydrogel of xylan‐*g*‐AGE‐DASH(A), domains with distinct mechanical characteristics were spotted over the mapping of 20×20 µm: soft domains with lower effective Young's modulus (marked by a red arrow in Figure 4f) and surrounding hard domains with higher effective Young's modulus. The xylan‐*g*‐AGE mesoglobules that were anchored in the polymeric network of PEG when photo‐crosslinked above the LCST are inferred to give rise to the comparatively soft domain. A heat map was plotted to showcase the spatial localization of effective Young's Modulus regions within these two hydrogels (Figure 4g,h), which presented distinct regions of high and low Young's modulus in xylan‐*g*‐AGE‐DASH(A), corresponding to the tough domain of PEGDA and soft domain of self‐assembled xylan‐*g*‐AGE. This material construction strategy leverages the bicontinuous structure, similar to those formed through polymerization‐induced phase separation.^[^ ^25^ ^]^

Nanoindentation reflects the local material mechanics on a microscale. Next, photorheology was employed to register the photocrosslinking kinetics of the hydrogel precursors during photopolymerization, and compression tests on the bulk hydrogels were employed to further determine whether the *TIPS* resulted in distinct mechanical properties at the macro‐scale between the homogenous hydrogel photocured below LCST and the biphasic hydrogel photocured above LCST. The effect of LCST of xylan‐*g*‐AGE on the phase separation of the photoresins was displayed in Figure S11 (Supporting Information). Photorheology was performed to ascertain the efficacy of the photopolymerization (i.e., free‐radical reactivity) among xylan‐*g*‐AGE, PEGDA, and 4‐arm PEGSH with different molecular weights, while xylan‐*g*‐AGE was present either as a solvated macromolecule or as self‐assembled mesoglobules in the photoresin. Figure S12 (Supporting Information) illustrates that the crosslinking of xylan‐*g*‐AGE below and above LCST upon photoinitiation at 63 and 87 s by UV_405_ shedding with a light density of 70 mW cm^−2^, respectively, and the photocrosslinking yielded a maximum storage modulus (*G′*, 6.4 kPa for xylan‐*g*‐AGE (B) and 18.6 Pa for xylan‐*g*‐AGE (A)) within 150 s, eventually fabricating an only xylan‐based hydrogel. This confirms that xylan‐*g*‐AGE is photopolymerizable either as a solvated macromonomer or as self‐assemblies. Notably, the crosslinking rate (defined as the required time from UV_405_ shedding to reach 90% of the maximum *G′*) of xylan‐*g*‐AGE (B) was higher than that of xylan‐*g*‐AGE (A), being 0.04 kPa s^−1^ compared to 0.0003 kPa s^−1^ for xylan‐*g*‐AGE (A), which was ascribed to the high transmittance and homogeneous state of photoresins below LCST.

**Figure 5:**
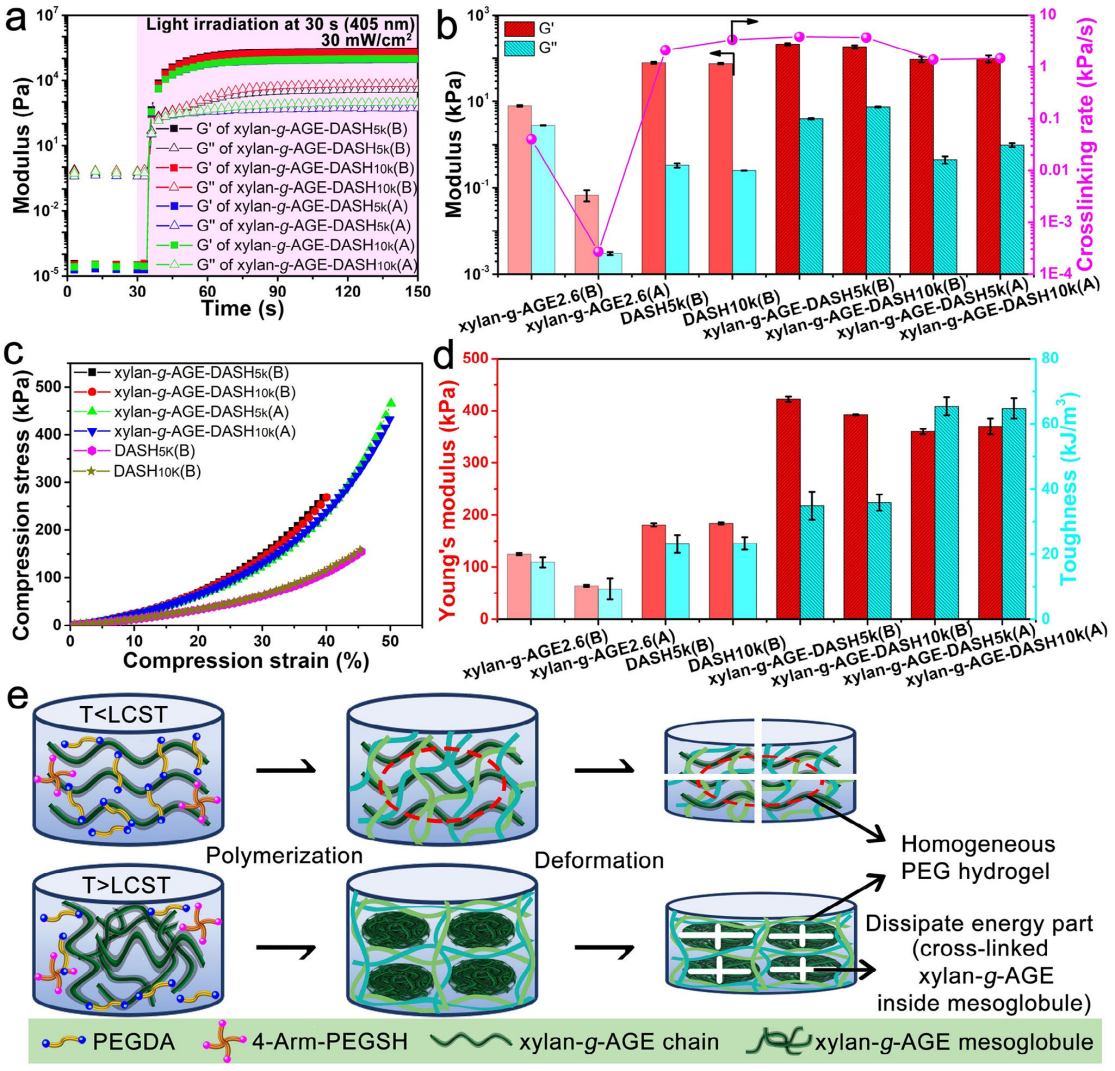
Xylan‐*g*‐AGE reinforced PEG hydrogel. a) Photo‐crosslinking profiles of xylan‐*g*‐AGE‐DASH hydrogel below and above LCST; b) Comparison of modulus and crosslinking rate of xylan‐*g*‐AGE and xylan‐*g*‐AGE‐DASH hydrogel below and above LCST; c) Stress–strain curves of the prepared hydrogels until fracture below and above LCST, n ≥5 independent samples; d) Comparison of Young's modulus and toughness of xylan‐*g*‐AGE and xylan‐*g*‐AGE‐DASH hydrogel below and above LCST; e) Schematic diagram illustrating the formation of homogeneous and heterogeneous hydrogel using xylan‐*g*‐AGE and PEGDA during the *TIPS* process below/above the LCST.

PEGDA is a long‐chain, hydrophilic, and cross‐linkable monomer, which has been widely used as a photoresin component for 3D hydrogel printing. To further enhance the toughness and increase the polymerization rate of PEGDA hydrogel, PEGSH was introduced to deploy thiol‐ene step‐growth polymerization, aiming to guarantee a more homogenous network in the hydrogel. As displayed in Figure S13 (Supporting Information), PEGSH could effectively improve the crosslinking rate of xylan‐*g*‐AGE hydrogel with UV irradiation of 30 kW cm^−2^, and the high molecular weight of PEGSH_10k_ increased the crosslinking rate to 0.081–0.085 kPa s^−1^, compared to 0.017–0.026 kPa s^−1^ of PEGSH_5k_. PEG‐DASH hydrogels present a higher crosslinking rate (2.1–3.3 kPa s^−1^) and *G′* (75.9–79.5 kPa) compared to the only xylan‐*g*‐AGE photoresin (Figure S12, Supporting Information). For the multicomponent photoresin of xylan‐*g*‐AGE‐DASH, the moduli of *G′* and *G′′* started to rise at ≈4 s and reached a plateau at ≈30 s after light shedding, as shown in **Figure** **5** **a**. The crosslinking rate of xylan‐*g*‐AGE‐DASH photoresins increased in the following order: xylan‐*g*‐AGE‐DASH_5k_(A) (1.4 kPa s^−1^) < xylan‐*g*‐AGE‐DASH_10k_(A) (1.47 kPa s^−1^) < xylan‐*g*‐AGE‐DASH_10k_(B) (3.66 kPa s^−1^) < xylan‐*g*‐AGE‐DASH_5k_(B) (3.8 kPa s^−1^) (Figure 5b). It is suggested that the incorporation of xylan‐*g*‐AGE does not significantly affect the crosslinking rate of the PEG‐DASH components. Though the hydrogels prepared below LCST exhibited higher crosslinking rate values than the hydrogel prepared above LCST, which reflects the possible light scattering effect from the *TIPS* of xylan‐*g*‐AGE on crosslinking rate.

The compressive stress–strain profiles of hydrogel discs photocured from various types of photoresins under different temperatures were compared in Figure 5c,d. As shown in Figures 5d and S14 (Supporting Information), xylan‐*g*‐AGE itself can be photo‐crosslinked to prepare only xylan‐based hydrogel with Young's modulus of 64–125 kPa and toughness of 9.3–17.5 kJ cm^−2^, which was lower than that of PEG‐DASH hydrogel, being 181–184 kPa for Young's modulus and 23.1–23.3 kJ cm^−2^ for toughness. When xylan‐*g*‐AGE was introduced as a solvated macromonomer below the LCST, it increased the compressive strength and stiffness of the hydrogel but decreased the compressive strain at break. Compared to the neat PEG‐DASH hydrogel, hydrogels of xylan‐*g*‐AGE‐DASH(B) exhibited ≈2.2 times higher Young's Modulus and ≈1.5 times higher toughness (Figure 5d). When xylan‐*g*‐AGE was introduced as self‐assemblies above the LCST, it simultaneously increases the compressive strength, the stiffness, and the compressive strain at break of the resulting hydrogels, and exhibits a superior toughening effect to the PEG‐based hydrogels. Notably, xylan‐*g*‐AGE‐DASH(A) exhibited ≈1.9 times higher Young's Modulus and ≈2.8 times higher toughness than the neat PEG‐DASH (Figure 5d). Interestingly, xylan‐*g*‐AGE exhibited different enhancement effects below/above LCST: xylan‐*g*‐AGE as a solvated macromonomer stiffens the PEG hydrogel stronger, while xylan‐*g*‐AGE as self‐assemblies toughens the PEG hydrogel better. The thermoresponsive self‐assemblies of xylan‐*g*‐AGE greatly improved the toughness of the photo‐crosslinked hydrogels, with maximal toughness up to ≈65.3 kJ m^−3^, compared to the hydrogel photocured with solvated xylan‐*g*‐AGE (toughness ≈34.9 kJ m^−3^). On one hand, the higher Young's modulus of xylan‐*g*‐AGE‐DASH(B) can be attributed to a larger amount of the AGE moiety available for denser crosslinking when xylan‐*g*‐AGE is present as solvated macromolecules, which also corroborates the faster crosslinking observed for xylan‐*g*‐AGE‐DASH(B) (Figure 5b). A homogeneous covalent interpenetration network was formed among solvated xylan chains and PEG chains, as illustrated in Figure 5e. On the other hand, the outstanding toughness of xylan‐*g*‐AGE‐DASH(A) originates from the biphasic but covalently bonded microstructures of soft domains of xylan‐*g*‐AGE mesoglobule dispersed in the hard domains of PEG matrix. The *TIPS* process above the LCST results in the dual chemically independent domains in the photoresin, and the photopolymerization ensures the connections through covalent bonding of thiol‐ene among the adjacent domains. In the opaque hydrogel, its biphasic microstructure combines the tough domain of PEG and the soft domain of self‐assembled xylan‐*g*‐AGE and ultimately improves their mechanical stiffness and robustness, as supported by the results in Figure 4f. The soft domain of xylan‐*g*‐AGE mesoglobules could enhance the stress transmission and diffusion when hydrogel discs of xylan‐*g*‐AGE‐DASH(A) were subjected to compression. The effective dissipation of energy within the bicontinuous network of distinct domains results in the hydrogel with exceptional mechanical strength, surpassing that of the homogeneous xylan‐*g*‐AGE‐DASH(B).

### 2.4 DLP‐Printed Conductive Hydrogel of xylan‐*g*‐AGE‐DASH@MXene in Wearable Strain Sensors

**Figure 6:**
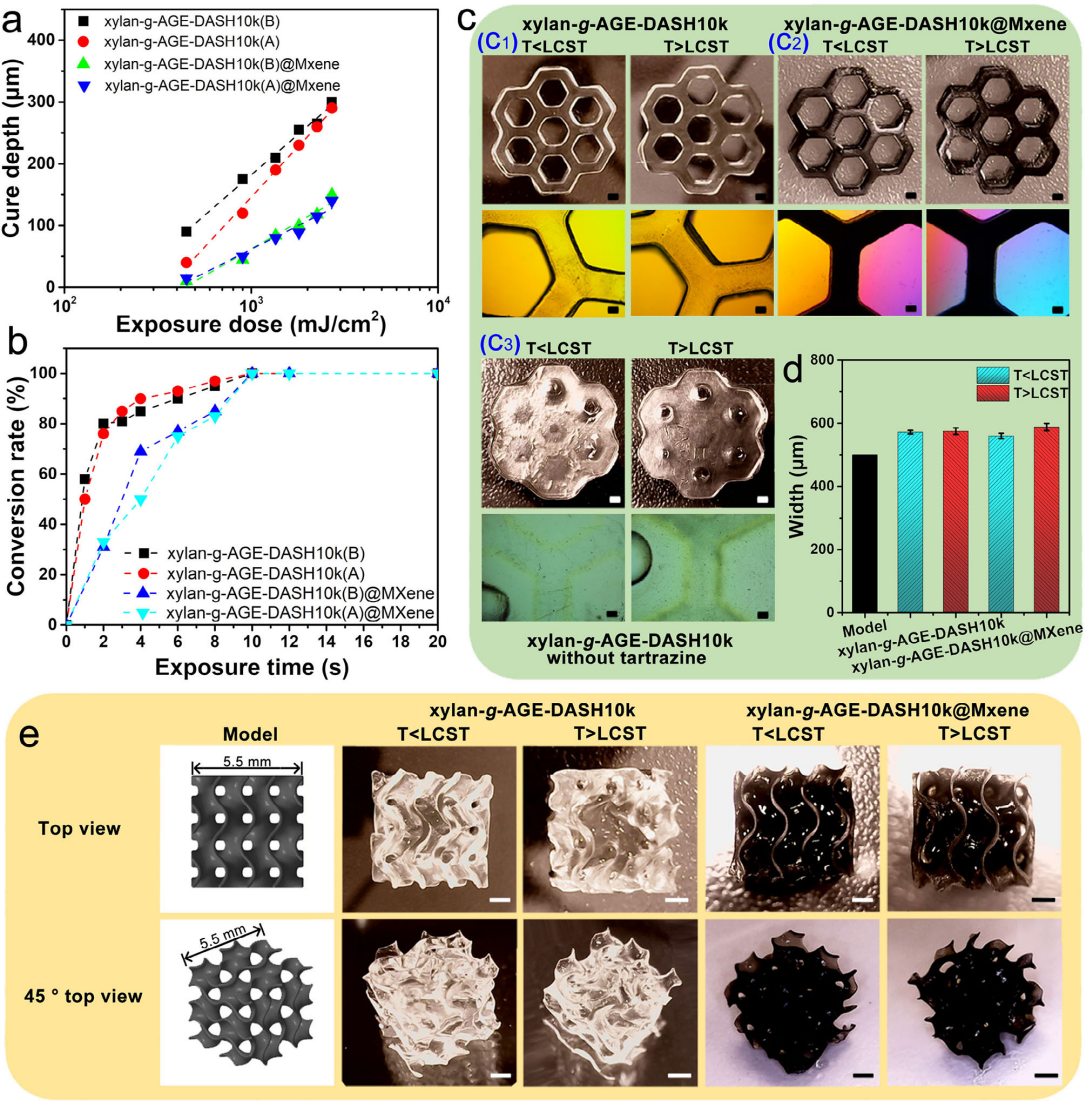
Photoinhibition mechanism of xylan‐*g*‐AGE and the printability of xylan‐*g*‐AGE‐DASH hydrogels. a) The cure depth of xylan‐*g*‐AGE‐DASH (with tartrazine) and xylan‐*g*‐AGE‐DASH@MXene (without tartrazine) photoresins with different exposure doses; b) The conversion rate of double bonds of photoresins with different UV shielding time; c) DLP printed honeycomb (scale bar is 500 µm) and corresponding optical microscope (scale bar is 200 µm) images; d) Comparison of the printing fidelity between designed model and hydrogels; e) Representative photographs of complex 3D structures (scale bar is 1 mm).

MXene sheets as a 2D material were introduced as a conductive element to further endow xylan‐reinforced PEGDA hydrogel with electric conductivity for utilization in smart devices. In photocuring upon UV irradiation, MXene sheets present strong light capture behavior originating from the electronic transitions and inherit a lamellar structure, which challenges the light penetration depth through the macromonomer solution and causes incomplete curing. This was observed when we tried to photocure a cast hydrogel (thickness above 1 mm) using the xylan‐*g*‐AGE‐DASH photoresin with the addition of MXene. In DLP printing, a hydrogel object is fabricated through layer‐by‐layer photocuring in the vat. During each printing step, a thin layer of the photoresin, with a controlled thickness (resolution of tens of microns), is confined between the building plate and the vat surface and subjected to photocuring. These technical features elegantly enable the 3D fabrication of hydrogels using photoresins such as the MXene‐containing xylan‐*g*‐AGE‐DASH. Prior to performing DLP printing, the cure depth and real‐time photo‐crosslinking kinetics of photoresins were determined to evaluate the photo‐absorbing effect of xylan‐*g*‐AGE and MXene. Above LCST, the cure depth of hydrogel is slightly lower than that prepared below LCST, most possibly due to poor light transmission in the opaque photoresin above LCST (**Figure** **6** **a**), which is consistent with the photo‐rheology results in Figure S12 (Supporting Information). Notably, Figure 6a indicates that MXene can function as a typical photo‐absorber, i.e., tartrazine, to reduce light scattering within the hydrogel precursor during the photocuring. A photo‐crosslinking rate analysis corroborated the findings in cure depth determination (Figure 6b). The photo‐crosslinking rate of xylan‐*g*‐AGE‐DASH hydrogel with tartrazine as a photo‐absorber occurred at a high rate compared to xylan‐*g*‐AGE‐DASH@MXene as the photoresin and the double bond conversion rate reached ∼100% within 12 s (see the detailed FTIR spectra in Supporting Information). In contrast, the photoresin of xylan‐*g*‐AGE‐DASH@MXene exhibited a slower crosslinking at the early stage (0–4 s), gradually accelerated within 4–8 s, and completely crosslinked by 12 s.

PEGDA‐based honeycomb hydrogel scaffolds containing xylan‐*g*‐AGE and MXene were fabricated via 3D DLP additive manufacturing (Figure 6c) to demonstrate printability. Based on the cure depth determined for PEGDA‐based hydrogel, the layer thickness, light intensity, and exposure time were determined to be 50 µm, 50 mW cm^−2^, and 12 s, respectively. PEGDA photoresins without tartrazine can be cast into hydrogels, but the propagated light to unirradiated regions simultaneously leads to blockage of the hollow structures (Figure 6c, 3). Thus, tartrazine as a soluble photo‐absorber or MXene was applied to suppress the scattering effect and improve the printing accuracy of DLP for photoresins, eventually fabricating a well‐structured honeycomb (Figure 6c1,c2). The toughening effect of xylan‐*g*‐AGE to the PEG network warrants the hydrogel fabrication with high robustness using biphasic photoresins via the *TIPS* process, supported by the results in Figure 5d, which would effectively enhance the adhesion between two adjacent layers and result in a better outcome of the building capacity on the Z axis for DLP printing. Moreover, PEGDA‐based hydrogels with different light transmittance were obtained due to the LCST behavior of xylan‐*g*‐AGE (Figure 6c, 1; Figure S15, Supporting Information). To determine the geometric discrepancy, a printing accuracy analysis was conducted by comparing the designed model and the actual width of the 3D‐printed honeycomb (Figure 6d). Compared to the model, PEGDA‐based hydrogel with good shape fidelity and printing accuracy over micrometers was obtained at both below and above the LCST.

The suppressed scattering effect of the photo‐absorber and improved toughness of the biphasic system (i.e., xylan‐*g*‐AGE and PEG‐DASH) allow the patterning of PEGDA‐based hydrogels to construct complex 3D structures. Based on the cure depth determined for PEGDA‐based hydrogel, the layer thickness, light intensity, and exposure time were determined to be 50 µm, 60 mW cm^−2^, and 10 s for xylan‐*g*‐AGE‐DASH_10k_, and 50 µm, 80 mW cm^−2^, and 12 s for xylan‐*g*‐AGE‐DASH_10k_@MXene, respectively. To guarantee a better adhesion between two adjacent layers, a higher curing depth (∼70 µm) was applied when compared to the layer thickness (50 µm). As displayed in Figure 6e, assisted by the computer‐aided design, highly porous and interconnective gyroid scaffolds (5.5 × 5.5 mm) with thin‐walled networks of 480 µm and a pore size of 330 µm were fabricated at both below and above LCST.

**Figure 7:**
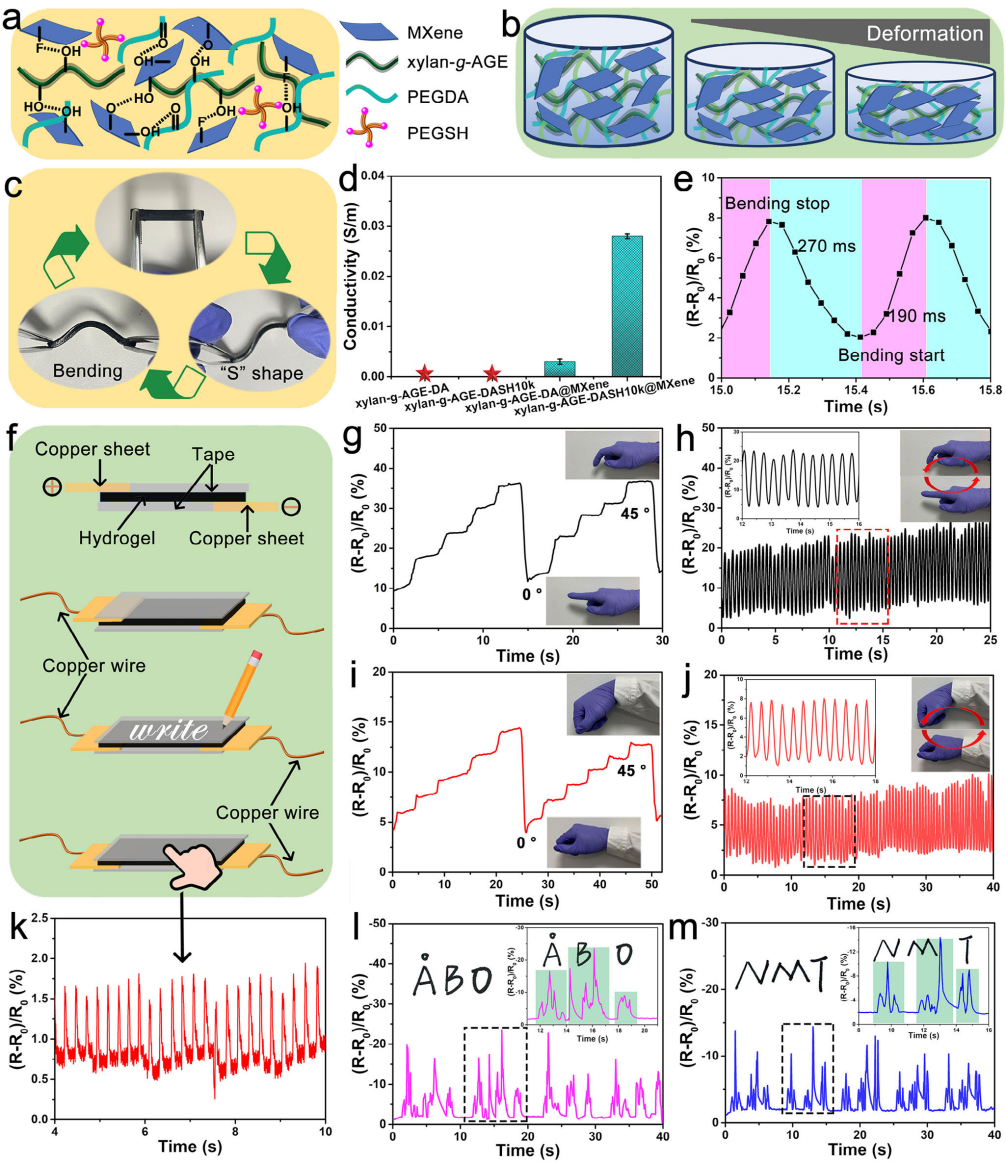
Conductive xylan‐g‐AGE‐DASH@MXene wearable strain sensors. a) The mechanism of better dispersion of MXene in the photoresins; b) Schematic illustration of the hypothesized resistance changes upon deformation; c) Optical pictures of the xylan‐*g*‐AGE@MXene hydrogel; d) The conductivity of various hydrogels; e) Response and recovery time of the xylan‐*g*‐AGE‐DASH@MXene hydrogel; f) Schematic illustration of xylan‐*g*‐AGE@MXene as a sensor or writing pad; Signals of relative electrical resistance during g,h) finger and i,j) wrist bending; k) Signals of relative electrical resistance as a pressure sensor; Signals of relative electrical resistance during writing l) “ÅBO” and m) “NMT”.

The single‐to‐few‐layered MXene sheet endows the hydrogel with good electrical conductivity after it's incorporated into xylan‐*g*‐AGE‐DASH hydrogels through the fabrication in DLP printing. We further demonstrated the possible utilization of these 3D‐printed conductive hydrogels in the field of wearable sensors below the LCST. As shown in **Figure** **7** **a**, rich functional groups (e.g., ─OH, ─F, and ─O) were introduced on the surface of MXene after etching and delamination, endowing the high hydrophilicity and extensive interfacial interaction of MXene, ultimately facilitating their dispersion and strong combination with photoresins. This was associated with the formation of H‐bonds between functional groups on the MXene surface and hydroxyl groups of xylan‐*g*‐AGE or carbonyl groups in PEGDA. Meanwhile, the conductive hydrogel can easily undergo deformations (e.g., bending and “S” shape) and return to its original shape after deformation, demonstrating the potential application in flexible wearable sensors (Figure 7c). During shape deformation, the spacing changes between MXene sheets changed the conductive pathway, simultaneously changing the corresponding resistance of the hydrogel (Figure 7b). The weak conductivity of xylan‐*g*‐AGE‐DASH hydrogel was mainly contributed by the H^+^ produced by water ionization (hardly to be detected), while the introduction of MXene greatly enhanced the electronic conductivity. Most importantly, the conductivity of xylan‐*g*‐AGE‐DASH@MXene hydrogel was higher (0.028 S m^−1^) than that of xylan‐*g*‐AGE‐DA@MXene (0.003 S m^−1^), which was DLP printed with photoink containing only xylan‐*g*‐AGE and PEGDA. Hydrogels of xylan‐*g*‐AGE‐DASH@MXene that deployed thol‐ene stepwise photopolymerization showed ≈9.3 times improvement in the conductivity, compared to the hydrogels of xylan‐*g*‐AGE‐DA@MXene that deployed free radical chain photopolymerization (Figure 7d). These results have suggested that the inclusion of PEGSH increases the crosslinking rate of photoresins (supported by the results in Figure 5b; Figure S12, Supporting Information), facilitating the dispersion of MXene and resulting in higher conductivity to the hydrogel. Above all, xylan‐*g*‐AGE‐DASH@MXene with fast response and recovery time (270 and 190 ms, respectively) could detect specific real‐time feedback signals, as shown in Figure 7e.

Given the high response/recovery time, high conductivity, and real‐time resistance change of xylan‐*g*‐AGE‐DASH@MXene hydrogel, they were assembled into wearable soft strain sensors and directly adhered to human skin to detect or monitor the real‐time movement of the body (Figure 7f). Prior to monitoring the body's movement, hydrogel of xylan‐*g*‐AGE‐DASH@MXene was attached to the knuckle/wrist to detect finger/wrist flexion increased from 0° to 45°, and the changes in current were recorded to calculate the corresponding relative resistance changes, which increased from a constant value to a specific value (Figure 7g,i). As shown in Figure 7h,j, stable and reproducible electrical signals can be detected from the movements of the finger and wrist. In addition, the hydrogel of xylan‐*g*‐AGE‐DASH@MXene exhibited a rapid and repeating responsive signal upon constant finger bending for 400 s, as shown in Figure S16 (Supporting Information). The electrical signals from real‐time detection can reflect the direction of motion, for instance, when the hydrogel was bent downward, a positive (R‐R_0_)/R_0_ value was obtained due to the increased resistance by elongation of the hydrogel. On the other hand, xylan‐*g*‐AGE‐DASH@MXene hydrogel also presented potential applications as pressure sensors, which were assembled with two copper films to form a “sandwich‐like” writing pad for signature recognition (Figure 7k). Taking advantage of rapid response time, a steady output of electrical signals can be monitored by pressing the hydrogel sensor with a finger. When “NMT” and “ÅBO” are written on the writing pad, writing motion/pressure‐induced resistance changes can be distinguished by the electrical signals. As presented in Figure 7l,m, different letters corresponded to different sharp and plateau peaks, so the letters can be accurately recognized by the specific electrical signals. As a result, xylan‐*g*‐AGE‐DASH@MXene hydrogel can monitor different signature patterns, showcasing the potential application in confidentiality and anti‐counterfeiting.

## 3 Conclusion

We have reported thermoresponsive and photoreactive xylan‐*g*‐AGE and validated its application in DLP 3D printing to toughen PEG hydrogel with high stiffness (422 kPa) and toughness (65.3 kJ m^−3^). Taking advantage of the D‐xylan of *β*‐1,4‐linked xylose possessing high flexibility and chemical reactivity compared to cellulose, thermoresponsive xylan‐*g*‐AGE with LCST property was fabricated via simple AGE modification, endowing free‐radical‐active and hydrophobic effects simultaneously. We demonstrated the possibilities to tailor the *T_cp_* of xylan‐*g*‐AGE by modulating the DS and concentration. The phase transition of xylan‐*g*‐AGE is associated with the reformation of H‐bonds and the hydrophobic effect in response to the temperature change. The thermoresponsive self‐assemblies of xylan‐*g*‐AGE reinforce the PEG hydrogels in photocuring, resulting in either transparent homogeneous hydrogel (i.e., solvated macromolecule of xylan‐*g*‐AGE photo‐crosslinked with PEG) below LCST or opaque heterogeneous hydrogel with bicontinuous morphologies (i.e., xylan‐*g*‐AGE mesoglobule domains dispersed in the PEG matrix) above LCST via *TIPS*. The material construction strategy of “dual chemically independent domains that are covalently bonded upon the thiol‐ene photopolymerization” is effective in terms of reinforcing the PEG hydrogel with greatly enhanced mechanical stiffness and toughness. The sustainable and thermoresponsive xylan‐*g*‐AGE, together with photoreactive PEGs, formulate a photoresin suitable for DLP printing to fabricate robust 3D hydrogel structures with complex geometry and high internal connectivity. The MXene sheet is a multifunctional additive that can be incorporated into photoresin to attenuate the light scattering in DLP printing and to endow the conductivity to hydrogel as wearable strain sensors. Our study comprehensively highlights the advantages of xylan‐*g*‐AGE with thermoresponsive self‐assembly behavior in toughening PEG hydrogel, demonstrating its potential for developing versatile, high‐performance, and sustainable xylan‐based materials through this design paradigm.

## 4 Experimental Section

### Materials

Allyl glycidyl ether (AGE, ≥ 99%), hydrochloric acid (HCl, 37%, ACS reagent), sodium hydroxide (NaOH, pellets, ≥ 99%), Titanium Aluminum Carbide 312 (powder, Ti_3_AlC_2_, ≥ 90%, ≤ 100 µm particle size), lithium fluoride (LiF, powder, <100 µm, ≥99.98% trace metals basis), Poly(ethylene glycol) diacrylate (PEG‐DA, average Mn 700), and tartrazine were purchased from Sigma–Aldrich. 4‐arm Poly(ethylene glycol) thiol (PEG‐SH, average Mn 5k, 10k) was purchased from biopharma PEG Scientific Inc.

### Synthesis of xylan‐*g*‐AGE

Native structure‐preserved xylan was extracted from birch using pressured hot water extraction.^[^ ^10^ ^]^ The linear D‐xylan was prepared by alkaline treatment at 150 °C for 8 h to remove side groups (e.g., 4‐O‐methylglucuronic acid) and acetyl groups of xylan. The reducing‐end groups of D‐xylan were transformed into primary alcohol using borohydride reduction to prevent the main‐chain peeling in alkaline conditions. The linear D‐xylan polymer was modified by an etherification reaction with AGE under alkaline conditions. Specifically, 4 g of freeze‐dried D‐xylan was mixed with 16 g of deionized water in a three‐necked flask under intensive stirring at 65 °C for 1 h. Then, 3.15 mL of NaOH (40 wt.%) was added, and the temperature was raised to 85 °C for the next 1 h. The flask was equipped with a water condenser and protected by a flowing N_2_ atmosphere. Afterward, 18 mL of AGE was added dropwise by an automatic pump device, and the reaction commenced for 24 h. After the modified D‐xylan was cooled and neutralized by formic acid (50 wt.%), a dialysis process was further deployed to remove impurities, and a transparent xylan‐*g*‐AGE dispersion was obtained.

### Preparation of MXene

MXene was synthesized by a minimally intensive layer delamination route using 12 m LiF and 9 m HCl.^[^ ^26^ ^]^ Briefly, 1.6 g LiF was added to 10 mL of 9 m HCl in an HDPE bottle with continuous stirring at 200 RPM for 5 min. Then, 1 g of Ti_3_AlC_2_ MAX powder was slowly added, and the stirring rate was increased to 400 RPM. After 24 h of the start of the reaction, the reactant solution was transferred into a centrifuge tube prefilled with DI water, and subsequently washed with DI water four times to neutralize at 3234 RCF for 5 min. The lithium intercalated MXene clay was mixed with 1 g LiCl and 50 g DI water in a plastic bottle under argon protection with intensive stirring at 65 °C for 1 h. After that, the intercalated solution was transferred into a centrifuge tube for washing. The vortex was used for each wash to ensure proper dispersion of the swelled MXene clay in water. After complete washing, set up a vortex for 30 min for the full dispersion of MXene in water. The resultant solution was centrifuged at 2380 RCF for 30 min to separate MXene clay from the single‐flake supernatant, which could be further bubbled with argon for prolonged storage.

### DLP Printing

The 3D printed models were designed by Autodesk Fusion 360 and sliced with X‐maker V2.7.1. M‐One Pro 30 DLP printer (Makex Co., Ltd, China) with HD UV‐LED (405 nm) light engine was used throughout the study. The detailed design of photoresins is listed in Table S2 (Supporting Information). Samples for curing depth measurements were prepared by injecting 100 µL of photoresins on top of a glass slide placed in the bath of the DLP printer. The photoresins were cured using different exposure doses varying between 150 and 900 mJ cm^−2^, and the cured thickness was measured using a 600×HD digital microscope. The light intensity was calibrated using a UV light meter probe (LS125‐UVALED‐Х3). After the printing process, the printed hydrogel was soaked in distilled water to remove the unreacted resin.

### Molecular Dynamics (MD) Simulations

The initial structure of the AGE‐modified D‐xylan was constructed using the Polymer Builder in Maestro Materials Science Suite (Schrödinger Release 2024‐3: Materials Science Suite, Schrödinger, LLC, New York, NY, 2024). The polymer repeat unit was built using the Monomer Sketcher by connecting five AGE molecules to two D‐xylose monosaccharides (Figure S17, Supporting Information). The monomer type was set to all‐atom, and the initiator and the terminator were defined as a hydrogen atom and a hydroxyl group, respectively. The composition of the polymer was set as a homopolymer with the degree of polymerization (DP) of 22 D‐xylose units and a total of 55 AGE‐molecules. The backbone dihedral was set to 180°. Two additional AGE‐molecules were inserted, and the ring structure at the end of the initial structure was opened with the 3D Builder. The final structure of the AGE‐modified D‐xylan had a degree of substitution of 2.6, containing 22 D‐xylose monomers and 57 AGE‐molecules. Due to the high computational cost when simulating the 22‐monomer long polymer, the polymer chain length was reduced to 10 monomers. The 10‐monomer chain model contained 26 grafted AGE‐molecules. The polymer model was structurally optimized using the semiempirical GFN2‐xTB method^[^ ^27^ ^]^ as implemented in the Jaguar Module of Maestro (Schrödinger Release 2024‐3: Semiempirical NDDO protocol; Jaguar, Schrödinger, LLC, New York, NY, 2024; MOPAC, Schrödinger, LLC, New York, NY, 2024). See the detailed MD simulations in the Supporting Information.

### Characterization—NMR Analysis

A Bruker AVANCE III 500 MHz NMR spectrometer equipped with a CryoProbe was applied to measure the liquid state ^1^H (338 K for 256 scans) and ^13^C (298 K for 12 000 scans) NMR of PHWE‐xylan, D‐xylan, AGE‐PHWE‐xylan, and xylan‐*g*‐AGE, respectively. Tetrabutylphosphonium acetate ([P_4444_][OAc]): DMSO‑d_6_ (1:4 / w:w) was used to dissolve samples with a concentration of 50 mg mL^−1^ before analysis. The DS of D‐xylan was calculated by comparing the integration of C═C double peaks (116.1 and 136.1 ppm) to anomeric carbons (C1, 101.5–103.4 ppm), according to Equation (1):

(1) $$DS=\frac{\left(A_{116}+A_{136}\right)/2}{A_{101}}$$

### Characterization—Rheological Analysis

The rheology properties of the photoresins were measured by an Anton Paar Multidrive rheometer (MCR 702, Anton Paar GmbH, Austria) using a parallel plate geometry (25 mm diameter) at 25 °C. The photo‐crosslinking kinetics of photoresins were determined under oscillation mode with a gap distance of 0.1 mm at a constant angular frequency and strain of 5 Hz and 0.5%, respectively. The photoresins were pre‐sheared at 100 s^−1^ for 20 s and equilibrated at 0.1 s^−1^ for 60 s before measurement. The photoresins were irradiated for 30 s by a light source (405 nm, bluepoint LED eco, Hönle Group, Germany) with a light density of 30 mW cm^−2^ through a transparent quartz bottom, and the change of *G′* and *G′′* was recorded.

### Characterization—Compression Test

The compression stress of photo‐crosslinked hydrogel (∼4.7 mm in diameter and 4.0 mm in height) was tested using a dynamic mechanical analyzer (MCR 702, Anton Paar GmbH, Austria) at a compression speed of 10 µm s^−1^. The initial elastic region of the compression stress–strain curves (10–15%) was used to calculate Young's modulus by linear fitting.

### Characterization—Nanoindentation Measurements and Analysis

The effective Young's modulus and topography of photo‐crosslinked hydrogel were measured by a Piuma Nanoindenter (Optics11 Life, Netherlands). For hydrogel measurement, 500 µL of photoresins were pipetted into a cylindrical container (Ø × H, 15 × 10 mm) and photo‐crosslinked using UV light. The probe used had 0.5 N m^−1^ and a spherical tip with a diameter of 3 µm. To minimize environmental errors, the measurements were performed in water with a loading rate of 2 µm s^−1^. Indentations for each sample were performed at an area of 20 µm in 10 × 10 square matrices for hydrogels.

### Characterization—Electrical Measurements

Electrical measurements were conducted using the Keithley 4200 A SCS Parameter Analyzer and a probe station to measure resistance data. The conductivity was calculated by the following Equation (2)^[^ ^28^ ^]^:

(2) $$\sigma =\frac{L}{R*S}$$

Where *L* is the distance (cm) between adjacent electrodes, and *σ*, *R*, and *S* are the conductivity (S/m), resistance (Ω), and cross‐sectional area (cm^2^) of the hydrogel, respectively.

The sensing performance of hydrogel was measured using a Keithley 4200 A SCS Parameter Analyzer and a probe station. The ends of the hydrogel samples (20 mm × 5 mm × 2 mm) were attached to conductive copper foil‐type tape, and the variation of resistance caused by bending or pressing the hydrogel was recorded. The relative resistance was calculated by the following Equation (3)^[^ ^28^ ^]^:

(3) $$\frac{\Delta R}{R_{0}}\;\left(\%\right)=\frac{R-R_{0}}{R_{0}}\;\times 100\%$$

Where *R* and *R_0_* represent real‐time resistance and initial resistance of the composite hydrogel, respectively. All participants signed the informed written consent prior to the research.

Detailed information regarding instrumental setups and experimental procedures for Fourier transform infrared (FTIR) spectroscopy, dynamic light scattering, turbiscan analysis, molecular weight determination, and thermogravimetric analysis (TGA) is listed in the Supporting Information.

## Conflict of Interest

There are no conflicts of interest to declare.

## Author Contributions

Y.Z. wrote the original draft, performed visualization, validation, methodology, investigation, formal analysis, and data curation. Q.W., W.D., S.H., and A.L. wrote, reviewed, and edited the final manuscript, performed methodology, formal analysis, and data curation. A.P. wrote, reviewed, and edited the final manuscript. performed validation, methodology, and investigation. O.M.H., S. A., and R.Ö. wrote, reviewed, and edited the final manuscript, performed validation, and gathered resources. C.X. wrote, reviewed, and edited the final manuscript, performed supervision, resources, investigation, and funded acquisition. X.W. wrote, reviewed, and edited the final manuscript, performed validation, supervision, project administration, methodology, investigation, conceptualization, and funded acquisition.

## Supporting information



Supporting Information

Supplemental Video 1

## Data Availability

The data that support the findings of this study are available from the corresponding author upon reasonable request.
