# Supplementary material for: “Grafting‐to” Polymers of Xylan‐g‐allyl Glycidyl Ether Toughen PEG Hydrogel via Microphase Separation: Thermoresponsive and Photoreactive Molecular Assembly in DLP 3D Printing
Source: Small. 2025 Jul 1;21(34):2502129. doi: 10.1002/smll.202502129 (PMC12393021; doi:10.1002/smll.202502129)
Supplement: Supplementary file 1 — Supporting Information [file SMLL-21-2502129-s001.docx]

**Supporting information**

**‘Grafting-to’ polymers of xylan-*g*-ally glycidyl ether toughen PEG hydrogel via microphase separation: thermoresponsive and photoreactive molecular assembly in DLP 3D printing**

*Yidong Zhang, Qingbo Wang, Wangfang Deng, Silva Hazer, Axel Luukkonen, Andrey Pranovich, Outi M. H. Salo-Ahen, Ronald Österbacka, Chunlin Xu, Xiaoju Wang ^*^*

Y. Zhang, Q. Wang, W. Deng, A. Pranovich, C. Xu, X. Wang

Laboratory of Natural Materials Technology

Åbo Akademi University

Henrikinkatu 2, FI-20500, Turku, Finland

Email: [xwang@abo.fi](mailto:xwang@abo.fi)

S. Hazer, O. Salo-Ahen, X. Wang

Pharmaceutical Sciences Laboratory

Åbo Akademi University

Tykistökatu 6A, FI-20520, Turku, Finland

S. Hazer, O. Salo-Ahen

Structural Bioinformatics Laboratory

Åbo Akademi University

Tykistökatu 6A, FI-20520, Turku, Finland

A. Luukkonen, R. Österbacka

Physics

Åbo Akademi University

Henrikinkatu 2, FI-20500, Turku, Finland

*Characterization*

*ATR-FTIR.* The attenuated total reflection Fourier transform infrared spectroscopy (ATR-FTIR) analysis of PHWE-xylan, D-xylan, AGE-PHWE-xylan, and xylan-*g*-AGE was performed on a Nicolet iS50 spectrometer (Thermo Fisher, New York, USA) in a wavenumber range of 4000−500 cm^−1^ with a spectral resolution of 4 cm^–1^.

*Dynamic Light Scattering.* The light scattering intensity of xylan-*g*-AGE was measured by dynamic light scattering (DLS) using a Zetasizer (Nano-ZS, UK). For the measurements, xylan-*g*-AGE at a concentration of 10 mg/mL was filtered through a 0.22 μm Nylon filter to remove large aggregates. The temperature was varied from 20 to 50 °C at increments of 3 °C and three measurements were made with a total of 15 scans at each temperature. The samples were pre-heated and equilibrated at a certain temperature for 3 min before each scanning.

*Turbiscan analysis.* The transmittance of the xylan-*g*-AGE solutions was measured using a turbiscan lab expert stability analyzer (Formulation) equipped with a pulsed near-infrared light source (λ = 880 nm) varying from 20 to 65 °C at increments of 3 °C. The samples were pre-heated and equilibrated at a certain temperature for 3 min before each scanning. The LCST was recorded as the temperature at which the sample's transmittance was 50%.

*TEM*. The morphologies of the xylan-*g*-AGE were observed using a TEM (JEM-1400 PLUS, Japan) at an accelerating voltage of 80 kV. The xylan-*g*-AGE (1 wt%) was heated up to 40 °C above LCST in an incubator, and then a small drop from the solution was deposited onto carbon-coated copper TEM grid at 40 °C above LCST. The excess xylan-*g*-AGE solution was sucked away by filter paper and dried in the incubator. The morphology of MXene suspensions was studied with a JEM-1400 PLUS TEM microscope (JEOL Ltd., Japan) with an accelerating voltage of 80 kV. Diluted sample (0.01 wt%) with 5 µL was deposited on the copper grid to wait for 3 min at environmental temperature. The excess of suspension was removed with filter paper prior to measurement.

*Molecular weight determination.* The molecular weight of PHWE-xylan, D-xylan, and xylan-*g*-AGE was measured using a high-performance size exclusion chromatograph equipped with an RI detector and a MALS detector (Wyatt Technology, USA). The samples were dispersed in water and washed with ethanol (99.5%) and DMAc three times to remove water. After that, the samples were dissolved in DMAc/LiCl (9%) to 20 mg/mL, then diluted three times with DMAC/LiCl (0.9 %) eluent before measurement. Two parallel analyses were carried out and the average value was reported.

*Thermogravimetric analysis.* The thermal stability of PHWE-xylan, D-xylan, AGE-PHWE-xylan, and xylan-*g*-AGE was performed on a thermogravimetric analyzer (TGA, SDT 650, USA) in the range of 30−600 °C with a heating rate of 10 °C/min under the protection of nitrogen (50 mL/min). The glass transition temperature (T_gDSC_) of PHWE-xylan, D-xylan, AGE-PHWE-xylan, and xylan-*g*-AGE was examined by DSC 25 (TA Instrument, USA) under the protection of nitrogen (50 mL/min) with the following heat-cool-heat sequence: 40 °C to 220 °C, 10 °C/min; 1 min for isothermal process; 220 °C to –80 °C, 10 °C/min; –80 °C to 220 °C, 10 °C/min. The T_gDSC_ of PHWE-xylan, D-xylan, AGE-PHWE-xylan, and xylan-*g*-AGE was calculated by the midpoint of the inflection transition half-height in the second heat capacity *vs.* temperature plot.

*Rheology analysis.* The viscosity of xylan-*g*-AGE was measured by an Anton Paar Multidrive rheometer (MCR 102, Anton Paar GmbH, Austria) using the bob/cup measuring system. The shear viscosity of xylan-*g*-AGE was monitored at the shear rate 10 s^-1^ at different temperatures.

*Microscopy Analysis.* A Nikon FN-S2N (Japan) microscope was used to image the xylan-*g*-AGE-DASH hydrogels. A LEO 1530 Gemini SEM (Zeiss/LEO, Germany) with an accelerating voltage of 2.70 kV was used to analyze the morphology of the MAX and MXene sheet.

*Conversion rate analysis.* The conversion rate of photoresin double bonds during photopolymerization was monitored by a Nicolet iS50 ATR-FTIR spectrometer (ThermoFisher Scientific). The uncured photoresin (~ 20 µL) was injected and confined using a glass coverslip, and then exposed to UV_405_ irradiation (light intensity 30 mW cm^−2^ at 1.5 cm above the sample) for a given time (0 – 20 s). After that, the IR spectrum was recorded immediately with a resolution of 4 cm^−1^ ranging from 4000 to 400 cm^−1^. The double bond conversion was calculated as Equation (1):

$$Conversion \left( \% \right)=(1-{{({A_{1415}}/{A_{1350}})}_{cured}}/{{({A_{1415}}/{A_{1350}})}_{uncured}})*100$$

Where, A_1415_ represents the absorption peak area of the in-plane scissoring vibration of ─CH acrylate end groups at 1415 cm ^−1^. The absorption peak area at 1415 cm ^−1^ is normalized to the absorption peak area at 1350 cm ^−1^.

*Molecular dynamic (MD) simulations*. To set up the simulation system in Maestro, an orthorhombic simulation box with periodic boundary conditions was created with the edge length of 193.552 Å. A randomized multi-component mixture was built using the Disordered System Builder in Maestro. The mixture consisted of the AGE-modified D-xylan (1.0 wt% = five 10-monomer chains) and water (99.0 wt%). The total number of molecules in the simulation system was 120000. We applied the OPLS4 force field ^[^[^1^](#_ENREF_1)^]^, and the initial state of the polymer was chosen to be a tangled chain, which is used for structures that have rotatable bonds. The Desmond MD engine ^[^[^2^](#_ENREF_2)^]^ as implemented in Maestro (Schrödinger Release 2024-3: Desmond Molecular Dynamics System, D. E. Shaw Research, New York, NY, 2024. Maestro-Desmond Interoperability Tools, Schrödinger, New York, NY, 2024) was used to carry out the simulations. The total simulation time of the production run was set to 200 ns and the energies and coordinates were saved every 200 ps to generate a simulation trajectory of 1002 frames. We used the isothermal-isobaric (NPT) ensemble where the particle number, pressure and temperature were kept constant during the simulation. Two simulations of the same simulation system were done using the same settings except for the temperature. The first simulation was done below LCST at 288.15 K and the second above LCST at 311.15 K. The pressure was kept at 1.01325 bar. The simulation systems were relaxed before the production simulation using Desmond’s default relaxation protocol (Bowers et al., 2006). The number of intermolecular and intramolecular hydrogen bonds and hydrogen bonds between the polymer and water were computed over the trajectory with the trajectory player.


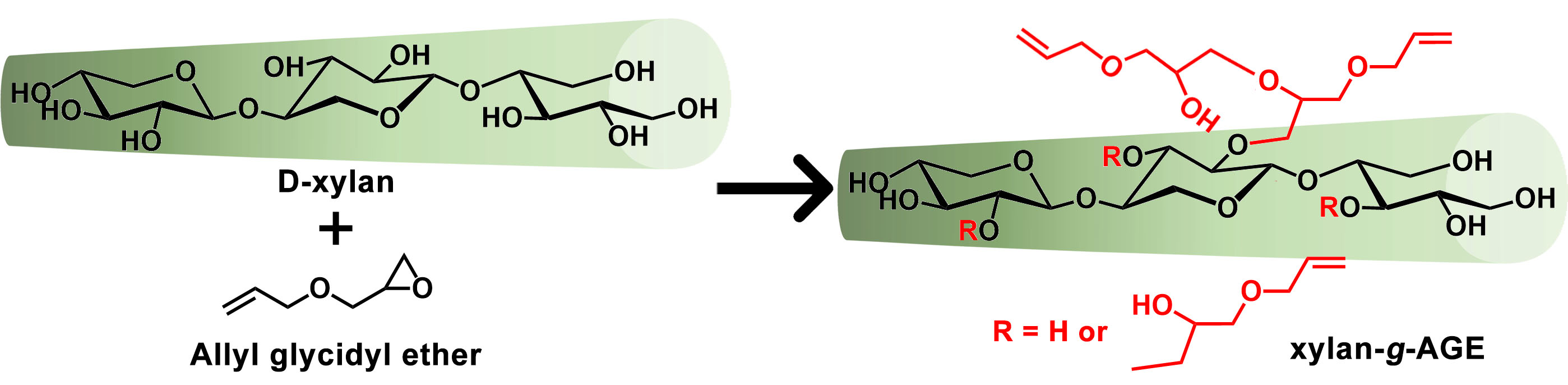


**Figure S1.** Schematic illustration of xylan-*g*-AGE with two AGE substituents after the etherification reaction.


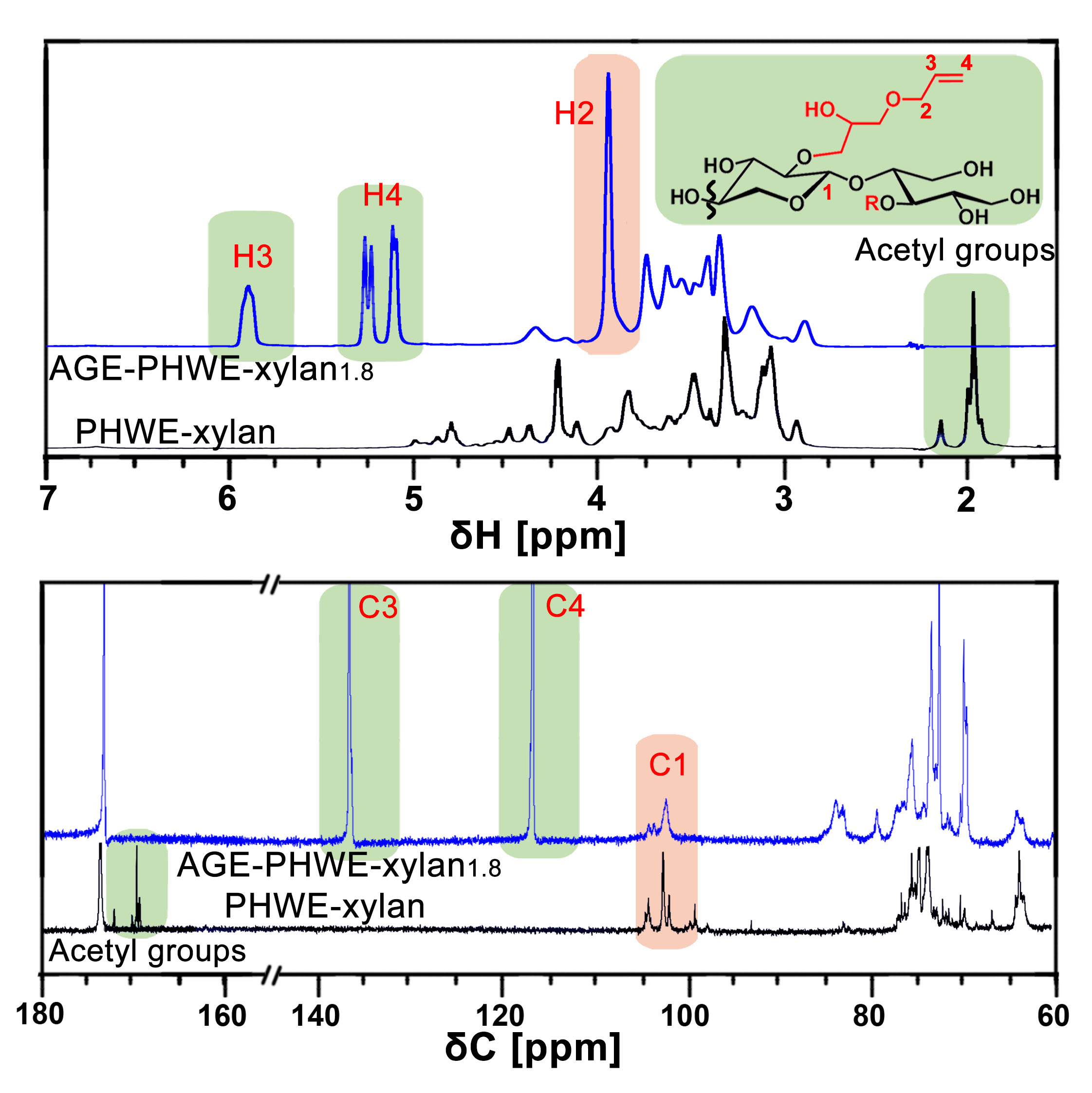


**Figure S2**. ^1^H NMR spectra and quantitative ^13^C NMR spectra of PHWE-xylan and AGE-PHWE-xylan_1.8_ as control (the used amount of NaOH (5.5 wt%) and the molar ratio of PHWE-xylan: AGE (1:5) was same as xylan-*g*-AGE_2.6_).


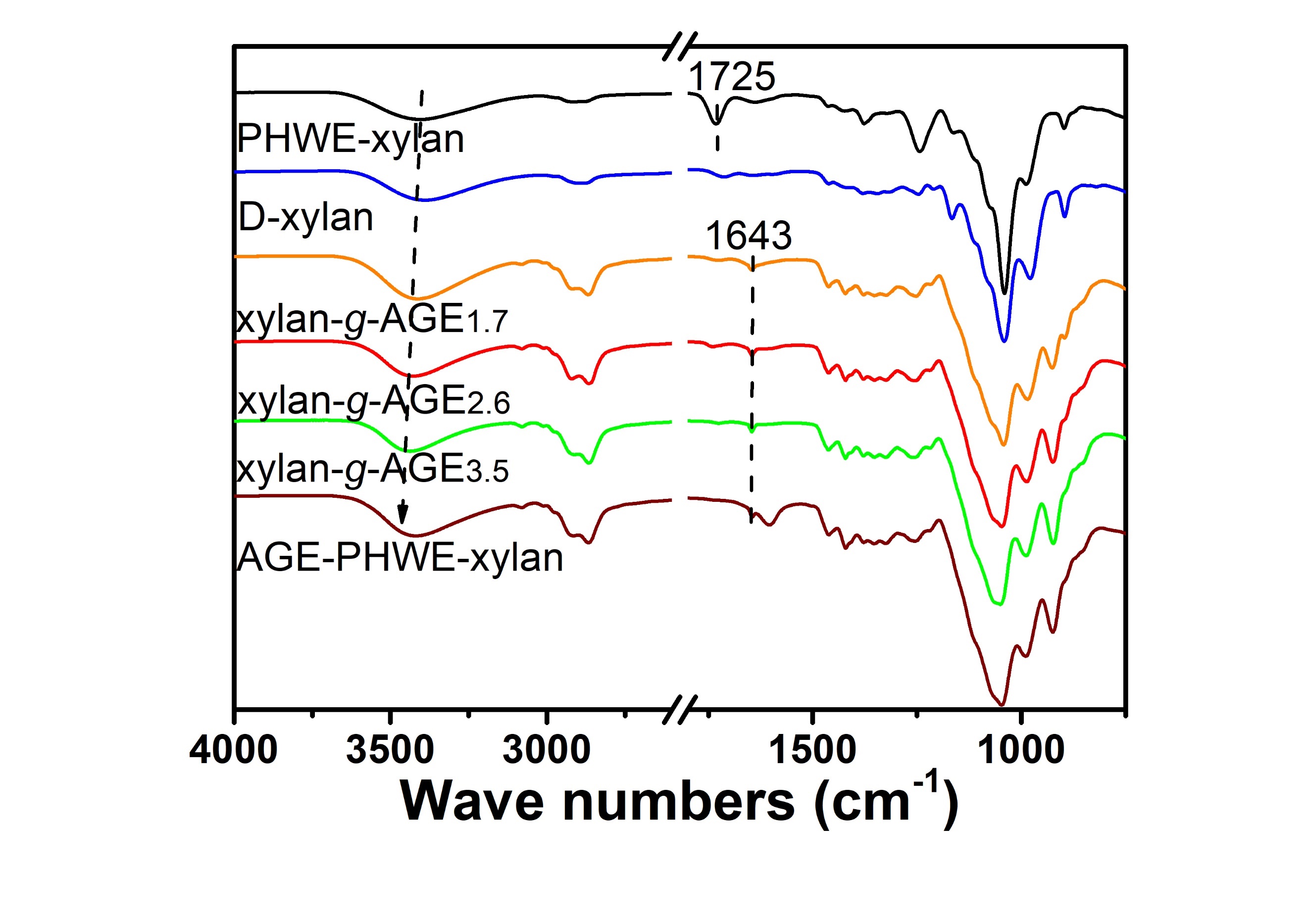


**Figure S3**. FTIR spectra of PHWE-xylan, D-xylan, AGE-PHWE-xylan, and xylan-*g*-AGE.


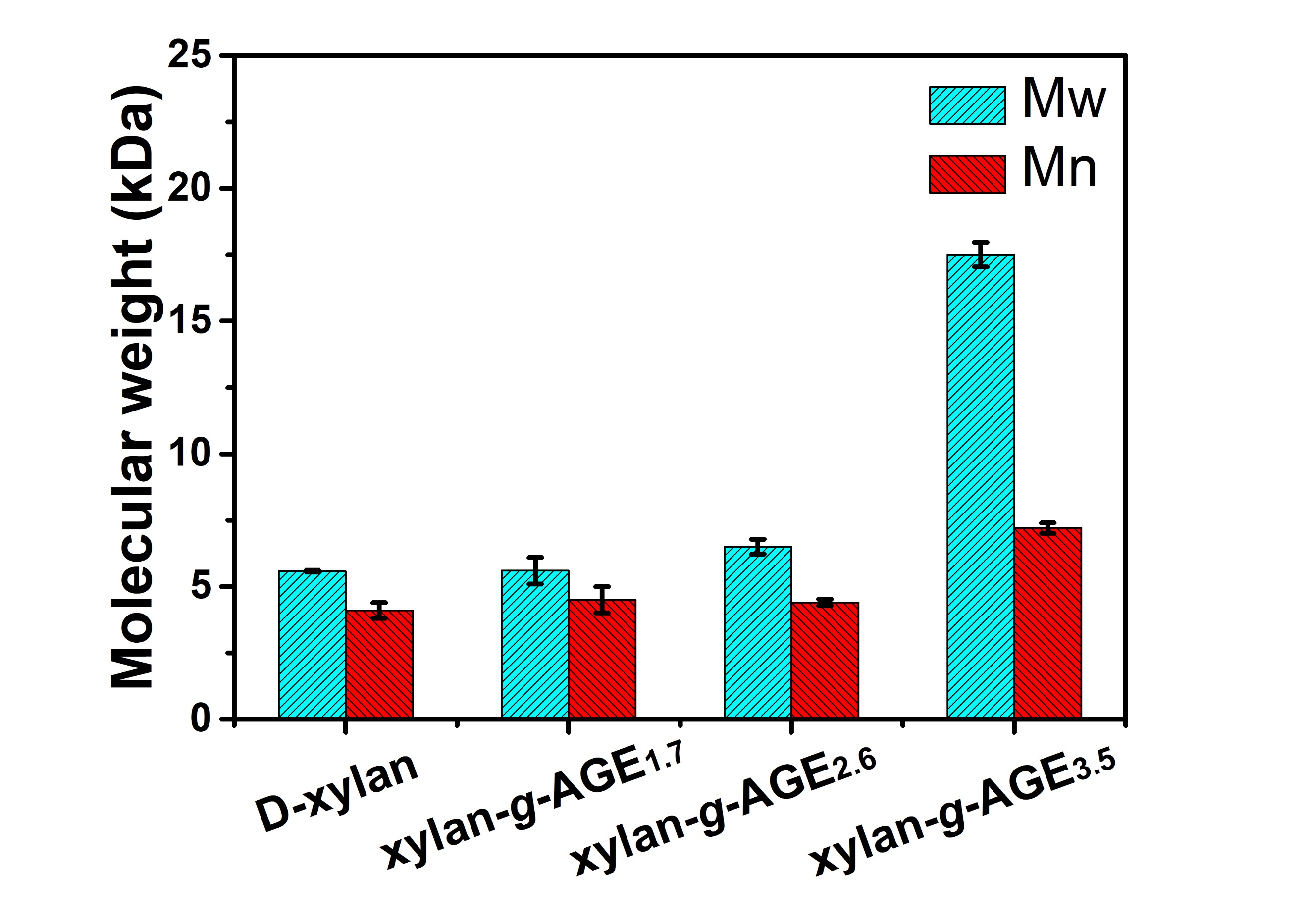


**Figure S4.** The molecular weight of D-xylan and xylan-*g*-AGE.


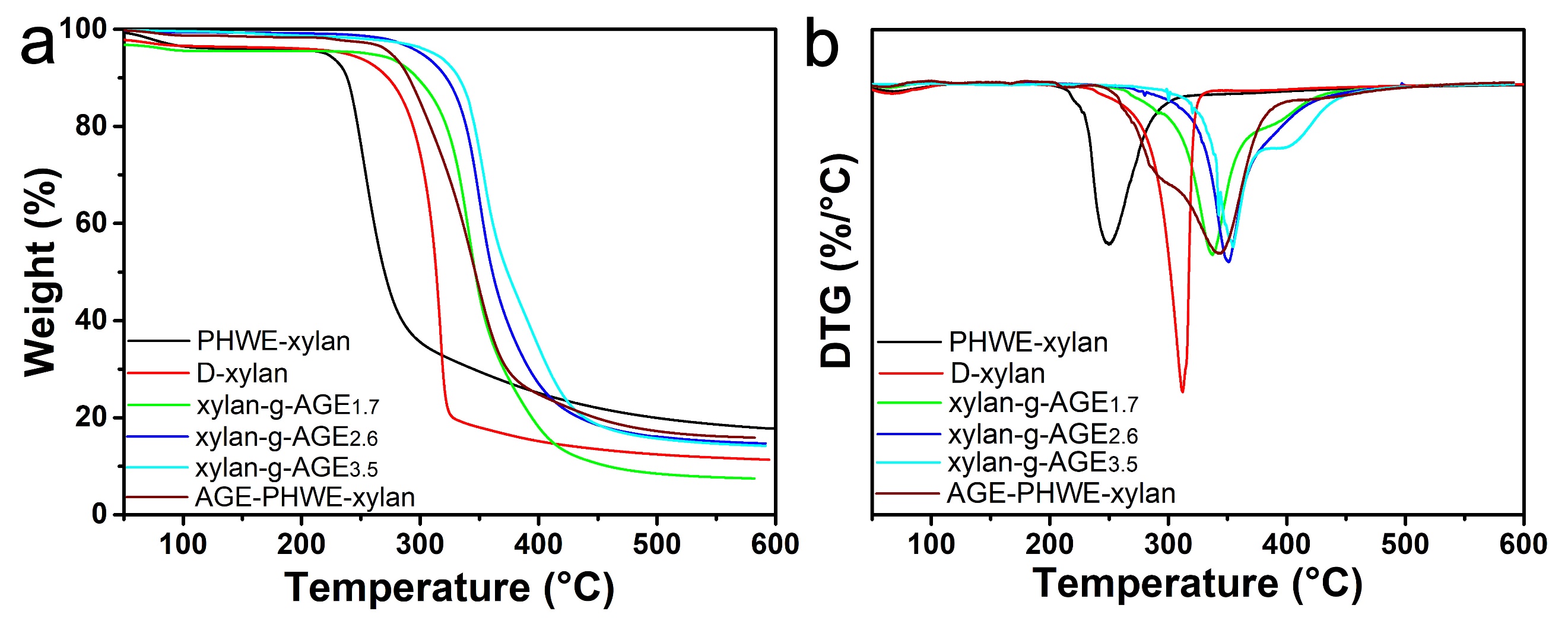


**Figure S5**. TG (a) and DTG (b) curves of PHWE-xylan, D-xylan, AGE-PHWE-xylan, and xylan-*g*-AGE.


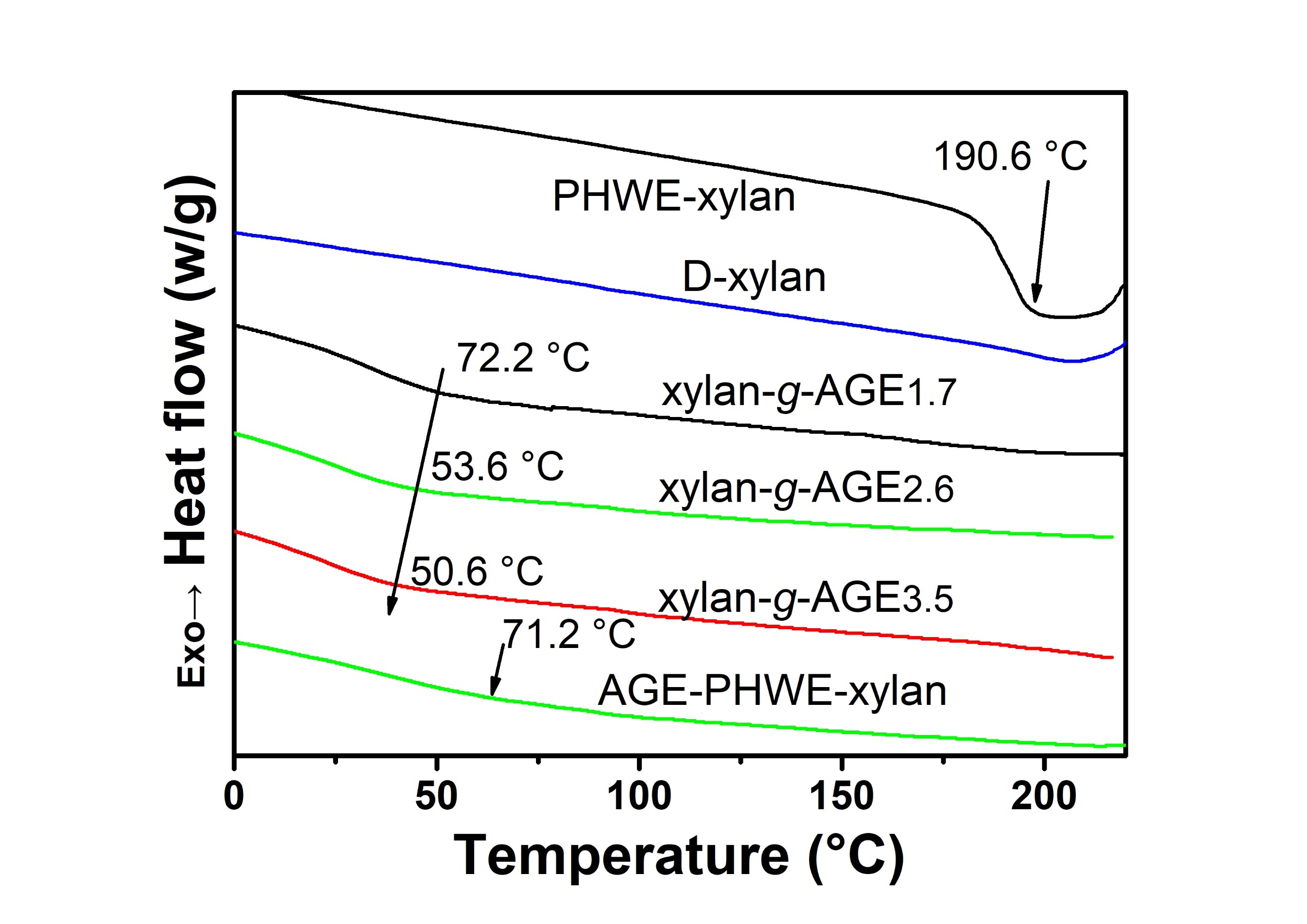


**Figure S6.** DSC curves of PHWE-xylan, D-xylan, AGE-PHWE-xylan, and xylan-*g*-AGE.


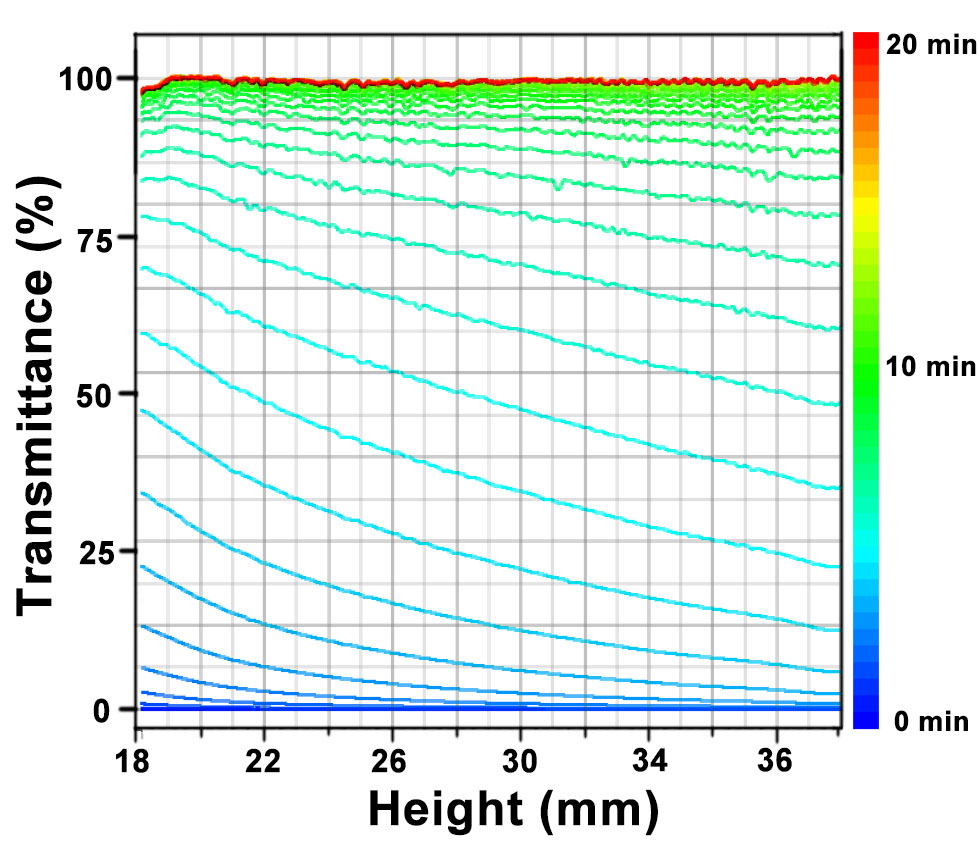


**Figure S7.** Transmittance of xylan-*g*-AGE changed over time. The set temperature of turbiscan was 25 °C, and xylan-*g*-AGE was heated above LCST.


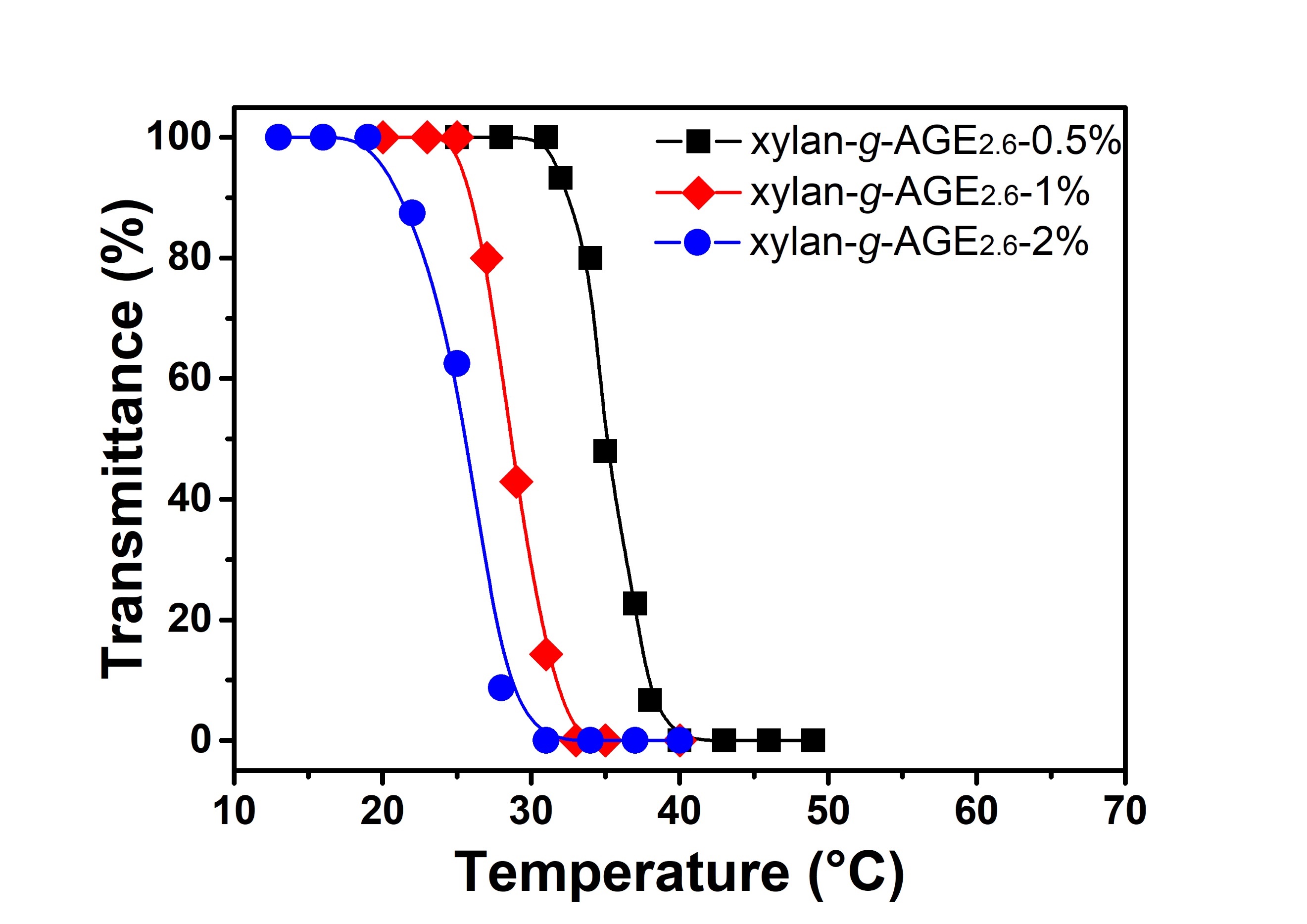


**Figure S8.** Diagram of transmittance vs. temperature of an aqueous xylan-*g*-AGE_2.6_ at different concentrations with full transmittance below the LCST followed by complete suppression above the LCST.


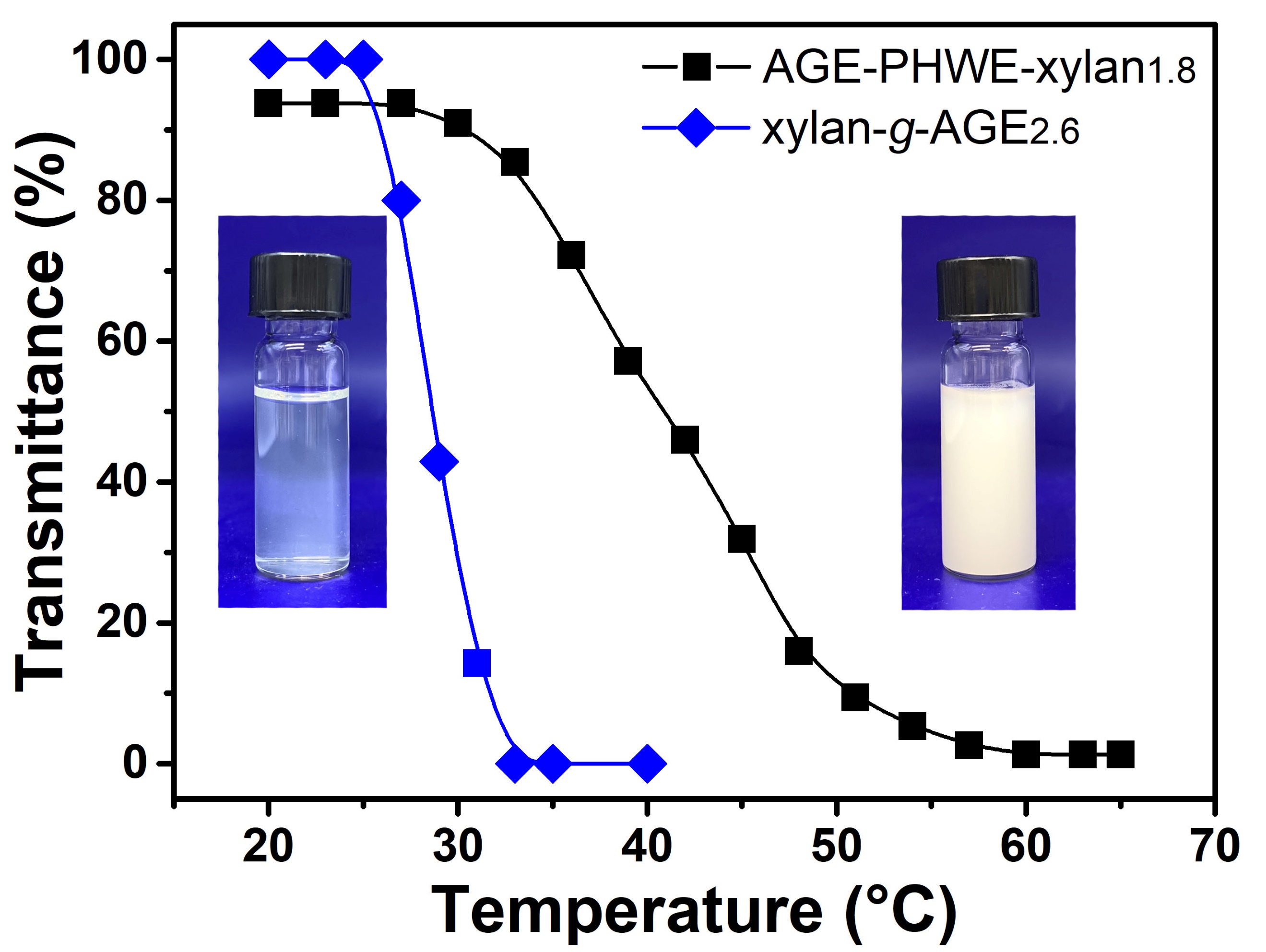


**Figure S9.** Diagram of transmittance vs. temperature of an aqueous AGE-PHWE-xylan_1.8_ (with side groups and acetyl groups as control with xylan-*g*-AGE) with full transmittance below the LCST followed by complete suppression above the LCST (Inset image is AGE-PHWE-xylan_1.8_).


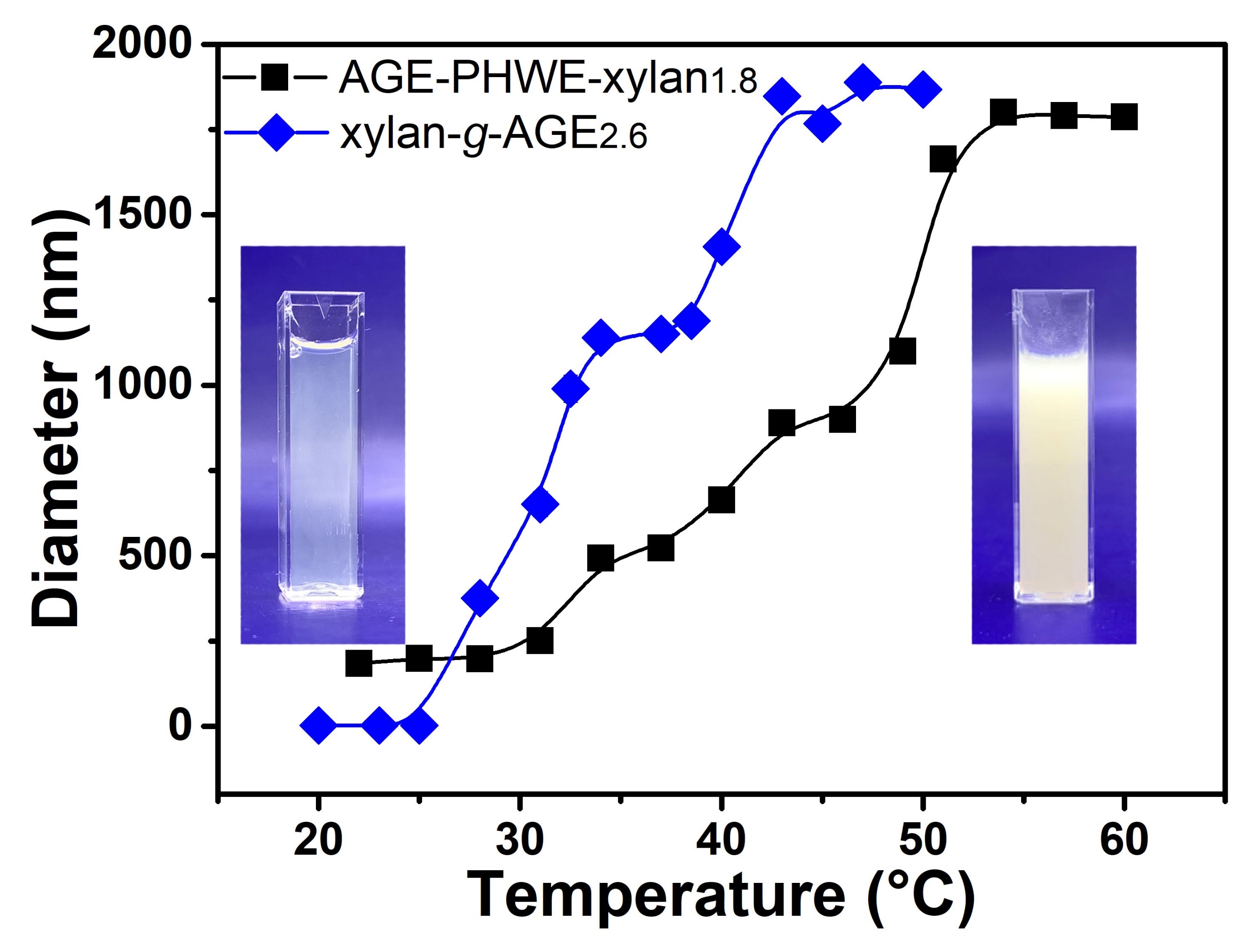


**Figure S10**. Diagram of hydrodynamic diameter vs. temperature of AGE-PHWE-xylan_1.8_ as control at different temperatures (Inset image is AGE-PHWE-xylan_1.8_).


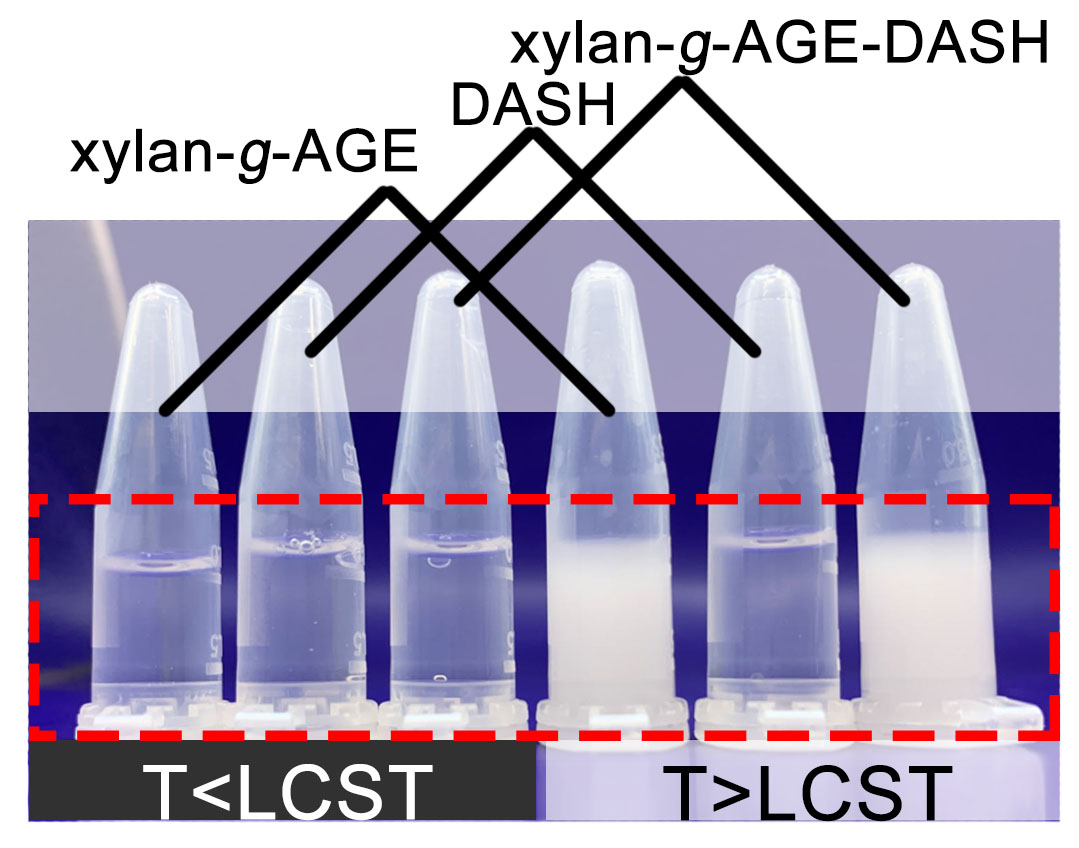


**Figure S11**. Photographs of different photoresins below/above LCST.


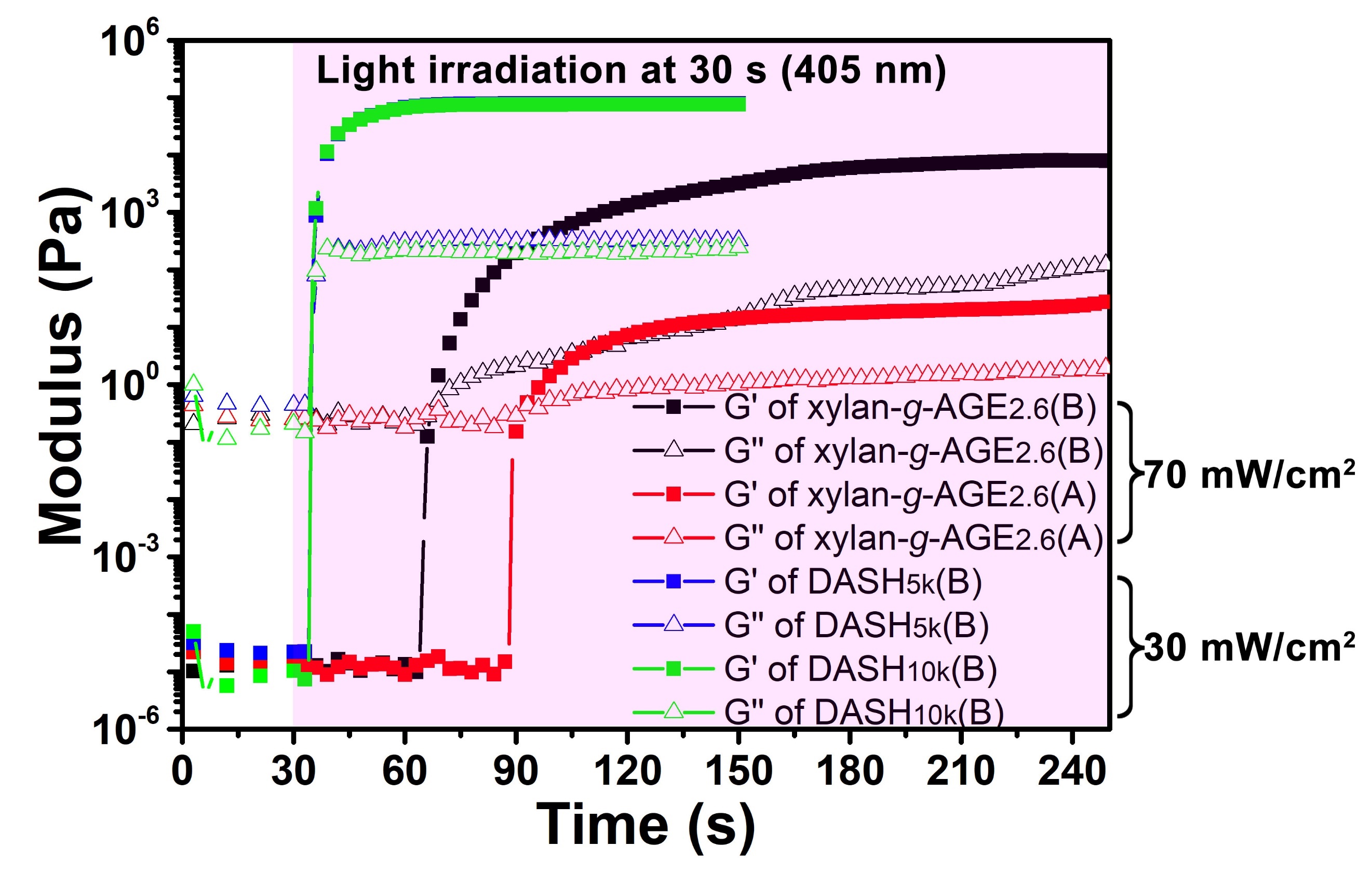


**Figure S12.** Photo-crosslinking profiles of xylan-*g*-AGE and DASH hydrogel below and above T_cp_.


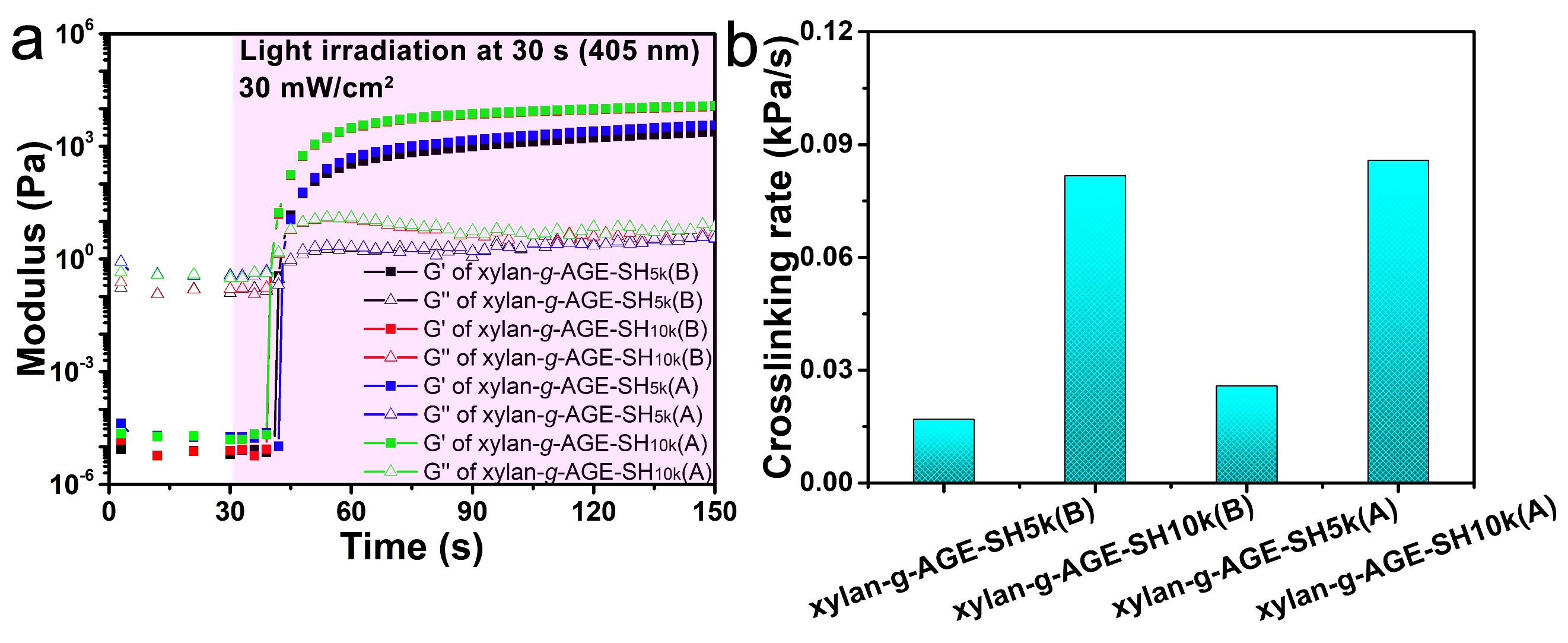


**Figure S13.** Photo-crosslinking profiles of xylan-*g*-AGE-SH hydrogel below and above T_cp_ (a); Comparison of modulus and crosslinking rate of xylan-*g*-AGE-DASH hydrogel below and above T_cp_ (b).


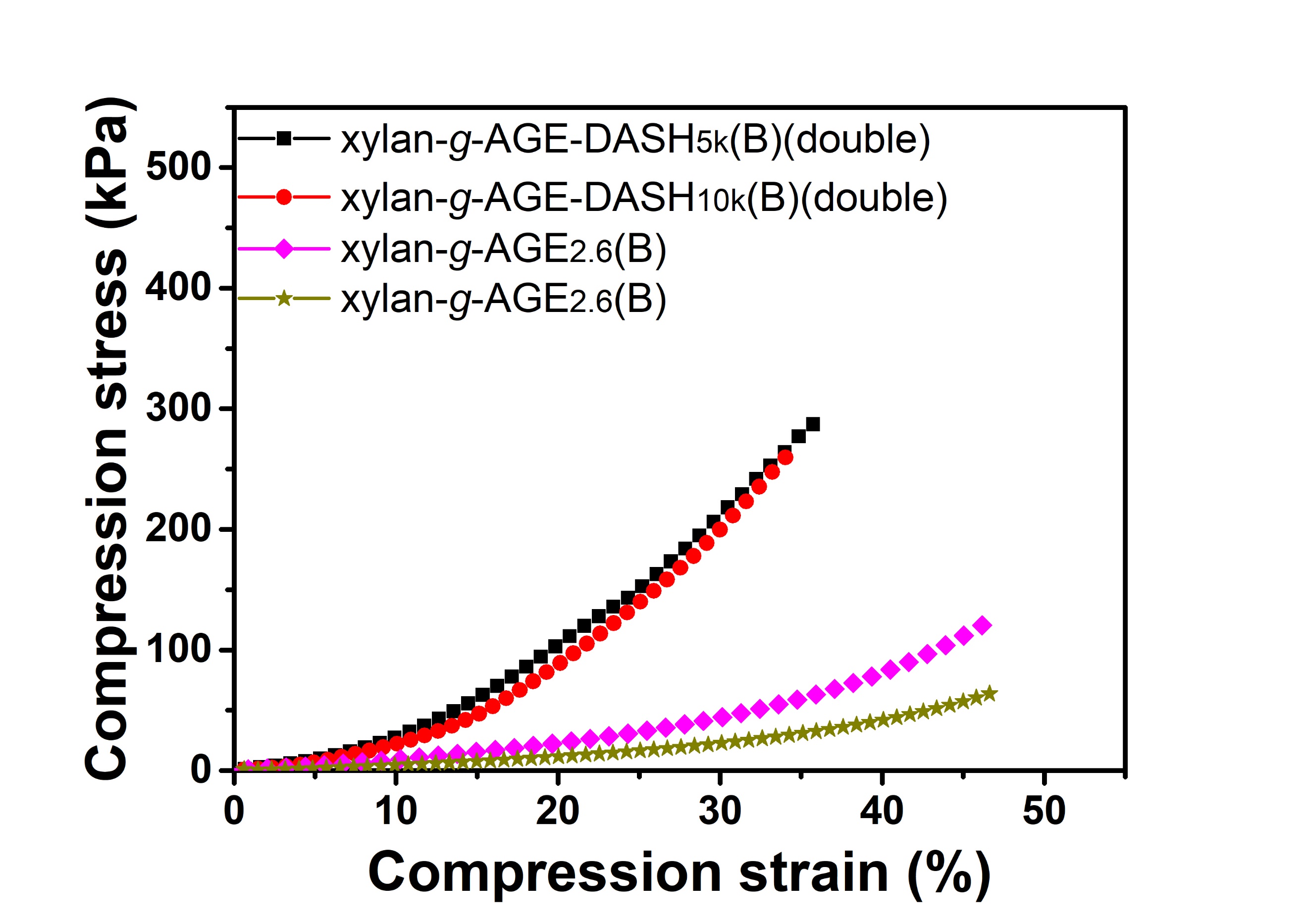


**Figure S14.** Stress-strain curves of the prepared hydrogels until fracture.


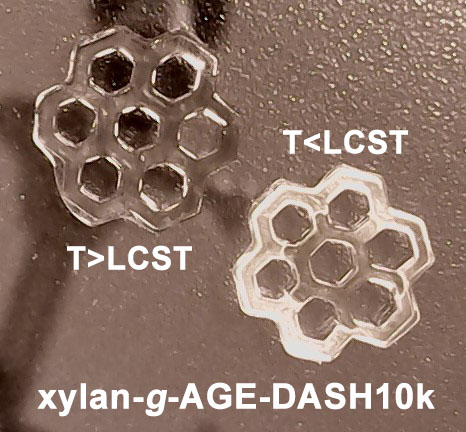


**Figure S15.** Photographs of xylan-*g*-AGE-DASH_10k_ hydrogel printed below/above LCST.


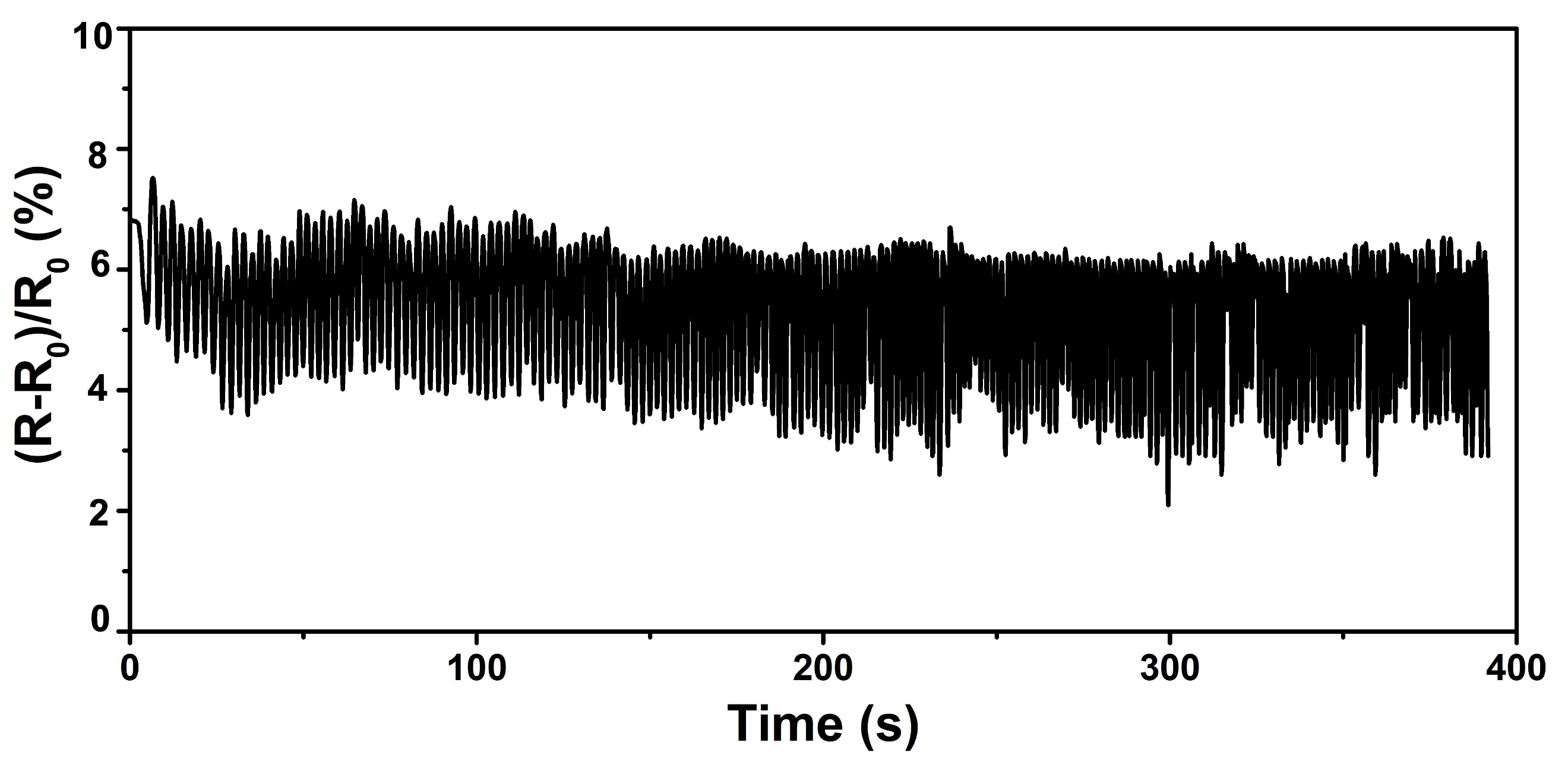


**Figure S16.** Signals of relative electrical resistance during finger bending for 400 s.


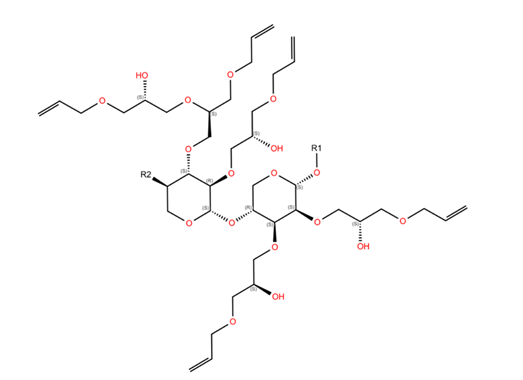


**Figure S17.** The structure of the polymer repeat unit used to build the 10-monomer xylan-*g*-AGE polymer for molecular dynamics (MD) simulations. The structure consists of two D-xylose monomers and five grafted AGE-molecules and has a *β*-1,4 linked xylose backbone.

**Table S1.** Thermogravimetric data of PHWE-xylan, D-xylan, AGE-PHWE-xylan, and xylan-*g*-AGE.

| Samples | PHWE-xylan | D-xylan | AGE-PHWE-xylan | xylan-*g*-AGE_1.7_ | xylan-*g*-AGE_2.6_ | xylan-*g*-AGE_3.5_ |
| --- | --- | --- | --- | --- | --- | --- |
| *T_o_* (°C) | 234.8 | 288.1 | 292.6 | 311.5 | 328.9 | 337.1 |
| *T_max_* (°C) | 250.3 | 309.7 | 342.9 | 337.2 | 350.1 | 354.1 |
| Residue (%) | 17.8 | 9.8 | 15.9 | 10.0 | 14.7 | 14.6 |

**Table S2.** Experimental design of photoresins.

| Samples | xylan-*g*-AGE_2.6_ (µL) ^a^ | 4-ArmPEG-SH (mg) | PEG-DA (mg) | LAP (µL) ^b^ | Tartrazine (µL) ^c^ | Water (µL) | MXene (µL) |
| --- | --- | --- | --- | --- | --- | --- | --- |
| xylan-*g*-AGE | 500 | 0 | 0 | 100 | 10 | 490 | 0 |
| xylan-*g*-AGE | 500 | 0 | 0 | 100 | 10 | 490 | 0 |
| xylan-*g*-AGE-SH_5k_ | 500 | 6.5 | 0 | 100 | 10 | 0 | 0 |
| xylan-*g*-AGE-SH_10k_ | 500 | 13 | 0 | 100 | 10 | 0 | 0 |
| DASH_5k_ | 0 | 35 | 100 | 100 | 10 | 790 | 0 |
| DASH_10k_ | 0 | 70 | 100 | 100 | 10 | 790 | 0 |
| xylan-*g*-AGE-DASH_5k_ | 500 | 35 | 100 | 100 | 10 | 290 | 0 |
| xylan-*g*-AGE-DASH_10k_ | 500 | 70 | 100 | 100 | 10 | 290 | 0 |
| xylan-*g*-AGE-DASH_10k_@MXene | 10 ^d^ | 70 | 300 | 100 | 0 | 0 | 500 ^e^ |

^a^ The concentration of xylan-*g*-AGE_2.6_ was 2 wt%; ^b^ The concentration of LAP was 2 wt%; ^c^ The concentration of tartrazine was 50 mM; ^d^ 10 mg of xylan-*g*-AGE was used; ^e^ the concentration of MXene was 2 wt%.

**References**

[1] C. Lu, C. J. Wu, D. Ghoreishi, W. Chen, L. L. Wang, W. Damm, G. A. Ross, M. K. Dahlgren, E. Russell, C. D. Von Bargen, R. Abel, R. A. Friesner, E. D. Harder, *J. Chem. Theory Comput.* **2021**, *17*, 4291-4300.

[2] K. J. Bowers, E. Chow, H. Xu, R. O. Dror, M. P. Eastwood, B. A. Gregersen, J. L. Klepeis, I. Kolossvary, M. A. Moraes, F. D. Sacerdoti, in *Proceedings of the 2006 ACM/IEEE Conference on Supercomputing*, **2006**, pp. 84-es.
